# Club cell CREB regulates the goblet cell transcriptional network and pro-mucin effects of IL-1B

**DOI:** 10.3389/fphys.2023.1323865

**Published:** 2023-12-20

**Authors:** Mariana Sponchiado, Angelina L. Bonilla, Luz Mata, Kalene Jasso-Johnson, Yan-Shin J. Liao, Amy Fagan, Victor Moncada, Leah R. Reznikov

**Affiliations:** Department of Physiological Sciences, University of Florida, Gainesville, FL, United States

**Keywords:** IL-1B, mucin, cystic fibrosis, club cell, FOXA2, SPDEF

## Abstract

**Introduction:** Club cells are precursors for mucus-producing goblet cells. Interleukin 1β (IL-1B) is an inflammatory mediator with pro-mucin activities that increases the number of mucus-producing goblet cells. IL-1B-mediated mucin production in alveolar adenocarcinoma cells requires activation of the cAMP response element-binding protein (CREB). Whether the pro-mucin activities of IL-1B require club cell CREB is unknown.

**Methods:** We challenged male mice with conditional loss of club cell *Creb1* and wild type littermates with intra-airway IL-1B or vehicle. Secondarily, we studied human “club cell-like” H322 cells.

**Results:** IL-1B increased whole lung mRNA of secreted (*Mucin 5ac, Mucin 5b*) and tethered (*Mucin 1, Mucin 4*) mucins independent of genotype. However, loss of club cell Creb1 increased whole lung mRNA of *member RAS oncogene family (Rab3D)*, decreased mRNA of the *muscarinic receptor 3 (M3R)* and prevented IL-1B mediated increases in *purinergic receptor P2Y, (P2ry2)* mRNA. IL-1B increased the density of goblet cells containing neutral mucins in wildtype mice but not in mice with loss of club cell Creb1. These findings suggested that club cell Creb1 regulated mucin secretion. Loss of club cell Creb1 also prevented IL-1B-mediated impairments in airway mechanics. Four days of pharmacologic CREB inhibition in H322 cells increased mRNA abundance of *forkhead box A2 (FOXA2)*, a repressor of goblet cell expansion, and decreased mRNA expression of *SAM pointed domain containing ETS transcription factor (SPDEF)*, a driver of goblet cell expansion. Chromatin immunoprecipitation demonstrated that CREB directly bound to the promoter region of *FOXA2*, but not to the promoter region of *SPDEF*. Treatment of H322 cells with IL-1B increased cAMP levels, providing a direct link between IL-1B and CREB signaling.

**Conclusion:** Our findings suggest that club cell Creb1 regulates the pro-mucin properties of IL-1B through pathways likely involving FOXA2.

## Introduction

Cystic Fibrosis (CF) is a life-shortening autosomal recessive genetic disorder caused by mutations in the *cystic fibrosis conductance regulator* (*CFTR*) gene. Adherent mucus and recurrent airway infections are responsible for most of the morbidity and mortality associated with CF. Mechanisms to explain adherent mucus are numerous (Mall et al., 2004; Pezzulo et al., 2012), and include hyperconcentration of the major gel-forming mucins, mucin 5B (MUC5B) and mucin 5AC (MUC5AC) (Abdullah et al., 2018).

Though goblet cells, and serous and mucous cells of the submucosal glands, are the major sources of mucins in the airways, club cells also secrete low levels (Hiemstra et al., 2015). Club cells are the major secretory cells in the small airways of humans, representing ∼15%–44% of all proliferating cells in terminal bronchioles (Boers et al., 1999). In mice, club cells represent greater than 50% of the total airway cells (Evans et al., 2004). Importantly, club cells can differentiate into goblet cells (Chen et al., 2009), and thus regulate the abundance of mucin in the airway. Conversion of club cells to goblet cells requires expression of SAM pointed domain containing ETS transcription factor (*SPDEF*) (Kim et al., 2019). In addition to *SPDEF*, forkhead box A2 (*FOXA2*) also plays a vital role in club cell differentiation and regulation (Chang et al., 2000; Chen et al., 2009). For example, *FOXA2* drives transcription of the club cell specific molecule secretoglobin 1A1 (SCGB1A1), also known as club cell secretory protein (CCSP) (Chang et al., 2000; Martinu et al., 2023) but represses mucin gene expression and goblet cell expansion (Wan et al., 2004). In this regard, SPDEF and FOXA2 are inversely related to one another (Chen et al., 2009; Chen et al., 2010; Yu et al., 2010; Choi et al., 2020). Though SPDEF suppresses FOXA2, it is not clear whether it is through direct or indirect mechanisms (Chen et al., 2009). Conversely, FOXA2 suppresses SPDEF, though it is likely indirect (Chen et al., 2010).

As highlighted earlier, club cells also express low levels of mucins under basal conditions, including MUC5AC (Evans et al., 2004) and MUC5B (Okuda et al., 2019). While club cell expression of these mucins can be increased in response to inflammatory cues (Evans et al., 2004; Rostami et al., 2021), MUC5AC is typically identified as an induced mucin (Evans et al., 2015), whereas MUC5B is constitutively expressed (Roy et al., 2014). However, overexpression of IL-1B in rodent airway club cells, for example, increases the number of airway mucus-producing cells (Bry et al., 2007). This is of interest given that IL-1B is the predominant cytokine in the human CF airway and correlates with airway mucin concentrations (Esther et al., 2019). Whether IL-1B converts club cells to goblet cells in human airways, and if so, whether that contributes to increased mucin concentrations in people with CF, is unknown.

The biological activity of IL-1B requires binding to the IL-1 receptor type I (IL1R1), leading to activation of several downstream signaling molecules, including the cAMP response element-binding protein (CREB) (Oger et al., 2002; Acuner Ozbabacan et al., 2014). In human airway epithelia, IL-1B increases cAMP through cyclooxygenase-2 and prostaglandin E2 pathways (Gray et al., 2004). A similar pathway has been described in airway smooth muscle (Misior et al., 2008). CREB is a transcription factor in the basic leucine zipper superfamily (Shaywitz and Greenberg, 1999) and a known regulator of *MUC5AC* (Song et al., 2003) and *MUC5B* (Choi et al., 2011) expression. Combined, these data shaped our hypothesis that the pro-mucin effects of IL-1B may require club cell CREB.

## Materials and methods

### Animals

Male and female mice heterozygous for floxed *Creb1* (030042-MU, B6N; B6J-*Creb1* ^tm1Nes^/Mmmh) were obtained from MMRRC Repository and bred to create homozygous progeny. Homozygous progenies were bred to mice purchased from the Jackson Laboratory (016225, B6N.129S6(Cg)-*Scgb1a1* ^tm1(cre/ERT)Blh^/J) containing tamoxifen inducible Cre-recombinase under the Secretoglobin Family 1A Member 1 (*Scgb1a1*) promoter. Scgb1a1, also known as clara cell secretory protein (CCSP), is expressed in club cells (Rawlins et al., 2009). The resultant heterozygous mice for each gene were crossed to produce mice homozygous for floxed *Creb1* (Creb1^fl/fl^) with (Scgb1a1^cre^) or without (Scgb1a1^WT^) inducible Cre expression in club cells. Mice with these genotypes (Creb1^fl/fl^Scgb1a1^cre^ and Creb1^fl/fl^Scgb1a1^wt^) were used for breeding up to 6 generations and maintained on a C57BL/6 background. Creb1^fl/fl^Scgb1a1^cre^ mice were crossed to Jackson Laboratory mice ROSA^mT/mG^ mice (strain 007576). Continual crossing occurred until mice homozygous for ROSA^mT/mG^ Creb1^fl/fl^Scgb1a1^cre^ and ROSA^mT/mG^ Creb1^fl/fl^Scgb1a1^wt^ were obtained (F4 generation). The genotypes were then mated to acquire mice that were ROSA^mT/mG^ Creb1^fl/fl^Scgb1a1^cre^ and ROSA^mT/mG^ Creb1^fl/fl^Scgb1a1^wt^ for study (F4 generation). All breeding was performed by the University of Florida Rodent Models breeding core. Adult (8–10 weeks old) male mice were studied. All mice were kept on 12 h light/dark cycle, fed *ad libitum* standard chow diet (2918, Teklad) and provided *ad libitum* access to water. Procedures were approved by and adhered to the University of Florida Institutional Animal Care and Use Committee. Strain details are provided in Supplementary Figure S1.

### IL-1B and tamoxifen treatment

Creb1^fl/fl^Scgb1a1^cre^ and Creb1^fl/fl^Scgb1a1^wt^ mice were lightly anesthetized under gaseous isoflurane (2%) in an induction chamber and intranasally administered 50 µL of sterile IL-1B (10 ng/mL) in 0.9% saline or sterile 0.9% saline vehicle using a pipette. This procedure occurred for four consecutive days. The rationale for this time course was as follows: 1) delivery of IL-13 in this manner leads to robust goblet cell hypertrophy in mouse lungs (Pezzulo et al., 2019); 2) increasing levels of IL-1B in the airway correlate with disease severity in mice post infection with influenza A virus (Bawazeer et al., 2021); and 3) in that study, the most severe disease occurred 4 days post infection, at which time 50% of the neutrophils expressed IL-1B (Bawazeer et al., 2021). All mice (Creb1^fl/fl^Scgb1a1^cre^ and Creb1^fl/fl^Scgb1a1^wt^) received an intraperitoneal injection containing 100 µL of sterile tamoxifen dissolved in corn oil (20 mg/mL) on days 1 and 3 to induce Cre-recombinase activity and excision of floxed *Creb1*.

### Recombination assessment

ROSA^mT/mG^ Creb1^fl/fl^Scgb1a1^cre^ (*n* = 3) and ROSA^mT/mG^ Creb1^fl/fl^Scgb1a1^wt^ (*n* = 2) mice were lightly anesthetized under gaseous isoflurane (2%) in an induction chamber and intranasally administered 50 µL of sterile 0.9% saline vehicle using a pipette. ROSA^mT/mG^ Creb1^fl/fl^Scgb1a1^wt^ (*n* = 2) were used as a negative control. This procedure occurred for four consecutive days. All mice received an intraperitoneal injection containing 100 µL of sterile tamoxifen dissolved in corn oil (20 mg/mL) on days 1 and 3. On day 5, mice were humanely euthanized using CO2 according to the AVMA guidelines, and their airways removed. OCT-embedded lungs were sectioned at 12 µm on a Microm HM 505 E cryostat and adhered to Superfrost plus microscope slides (Fisher Scientific). Sections were then fixed in 2% paraformaldehyde and prepared for antibody labeling using general procedures previously described by our lab (Kuan et al., 2019). Sections were incubated with SCGBA1/CCSP antibody (rabbit anti-CCSP; 07-623, Millipore Sigma; 1:1000 dilution; 2 h). Secondary antibody labeling was accomplished using alexa fluor goat anti-rabbit 350 (Life Technologies, cat. #A11046, 1:1000 dilution, 1 h). Images were assessed using ImageJ. A mask was created for the blue channel (representing CCSP) and green channel (representing GFP due to recombination) and the overlap of the area of the green mask with the area of the blue mask for 2- 6 airways was determined and reported as a percent average. Prior work from the original characterization paper of *Scgb1a1* ^tm1(cre/ERT)Blh^/J mice suggested that a two dosing regimen of tamoxifen resulted in detectable Cre recombination in 50%–60% of the bronchiole club cells (Rawlins et al., 2009). We found recombination rates in the airway labeled with CCSP to be ∼75% (Supplementary Figure S3).

### FlexiVent

Pulmonary mechanics were evaluated 20–24 h after the last IL-1B administration. Procedures were performed as previously described (Reznikov et al., 2018). Briefly, a tracheotomy was performed in anesthetized mice (ketamine/xylazine/acepromazine) and a cannula (blunted 18g needle) was inserted into the trachea. Mice were ventilated at 150 breaths/min at a volume of 10 mL/kg of body mass and administered a paralytic (rocuronium bromide). Increasing doses of methacholine from 12.5 to 100 mg/mL (Reznikov et al., 2018) were aerosolized using an ultrasonic nebulizer. Anesthetized animals were euthanized at the end of the flexiVent protocol via cervical dislocation.

### Bronchoalveolar lavage (BAL) and analyses

Three sequential 1 mL lavages of 0.9% sterile saline were delivered into the airway *postmortem*. All collected material from one mouse was pooled, spun at 500 x g, and the supernatant removed and frozen at −80°C. Cells were counted on a hemocytometer.

### Enzyme-linked immunosorbent assay (ELISA)

ELISAs for murine MUC5AC (M7906) and murine MUC5B (M7978) were purchased from Biotang (Lexington, MA, US). BAL samples were run in duplicate and read using a filter-based accuSkan FC microplate photometer (ThermoFisher Scientific, Waltham, MA, US). Concentrations were determined by an 8-point standard curve ranging from 0.625 to 80 ng/mL plotted in a 4-parameter logistic sigmoidal curve (R^2^ > 0.99). MUC5AC and MUC5B protein standards were provided in the kits. The limits of sensitivity were 0.3 ng/mL for both. The intra-assay coefficients of variability were 8.89% and 6.88% for MUC5AC and MUC5B, respectively. An ELISA for IL-13 (M1300CB) was purchased from R&D Systems (Minneapolis, MN). Concentrations were determined by an 8-point standard curve with ranges of 7.8–250 pg/mL and plotted in a 4-parameter logistic sigmoidal curve (R^2^ > 0.99). The intra-assay and inter-assay variabilities were 2.8% and 3.6%, respectively.

### Histology

Following flexiVent procedures and exposure to the methacholine dose response curve, the left lung was removed and placed in 10% normal buffered formalin. An additional set of mice did not undergo flexiVent and were humanely euthanized by CO2 according AVMA guidelines. Their left lung was also removed and placed in 10% normal buffered formalin. Lungs were not pressure inflated or actively perfused with fixative. Standardized procedures were established in which lungs were embedded in paraffin and transversely sectioned starting at airways most distal to the bronchus. All lungs were embedded and sectioned in standardized fashion with the ventral lobar surfaces oriented down in the cassette. Paraffin-embedded samples were sectioned at 4 μm thickness transversely through the terminal bronchioles and lower bronchioles. Sections containing lower bronchioles were selected and stained with Alcian Blue/Periodic Schiff (PAS stain) (Epredia, cat. #87023), according to the manufacturer’s instructions. Airways were imaged using a Zeiss Axio Zoom.V16 (Carl Zeiss, Germany) microscope. Alcian Blue/PAS-positive cells were counted independently by two masked observers and normalized to airway luminal area using ZenPro software (Carl Zeiss). Alcian Blue/PAS-positive cell numbers were averaged across observers and the mean per mouse was used for statistical analysis.

### Immunohistochemistry

Paraffin-embedded lung sections (4 μm) were deparaffinized and endogenous peroxidase activity blocked with 3% hydrogen peroxide in methanol for 30 min. Sections were incubated in 10 mM sodium citrate buffer (pH 6) and microwaved for antigen retrieval. MUC5B (rabbit anti-MUC5B; HPA008246, Millipore Sigma; 1:500 dilution; 2 h) immunolabeling was performed using the Vectastain Elite ABC system (PK-6101, Vector Laboratories, CA, US) according to the manufacturer’s protocol. Staining was developed with 3,3′-diaminobenzidine tetrachloride (34065, ThermoFisher Scientific). Samples were counterstained with hematoxylin. Images were captured with a Zeiss Axio Zoom.V16 (Carl Zeiss) microscope. MUC5B mean intensity in central airways was semi-quantified by tracing the epithelial area and using the IHC plugin in ImageJ (http://rsb.info.nih.gov/ij/). Dual labeling for CCSP (rabbit anti-CCSP; 07-623, Millipore Sigma; 1:1000 dilution; 3 h), a marker of club cells, and Creb1 (mouse anti-Creb1; MA1-083, Invitrogen; 1:200 dilution; 3 h) was achieved using the ImmPRESS Duet Double Staining Polymer Kit (MP-7724, Vector Laboratories). A mouse-on-mouse blocking step (MKB-2213-1; Vector Laboratories) was added. The proportion of CCSP-positive cells in the murine airway that were also positive for Creb1 was assessed by two independent masked observers. Cell numbers were averaged across observers and the mean per mouse was used for statistical analysis.

### Cell culture and treatment

Human NCI-H322 cells were obtained from the European Collection of Authenticated Cell Cultures (ECACC; Sigma). Ultrastructural studies of this male bronchoalveolar adenocarcinoma-derived cell line demonstrated the presence of cytoplasmic structures characteristic of club cells (Lau et al., 1987; Schuller et al., 1991). NCI-H322 cells were grown in RPMI medium (11875-093; Gibco) supplemented with 10% fetal bovine serum (26140-079; Gibco) and 1% penicillin/streptomycin (15140-122; Gibco). Cultures were maintained at 37°C and 5% CO_2_.

A) IL-1B treatment and CREB inhibitor experiment. Cells (passage 105) were seeded onto 12-well plates. At subconfluency (90%), monolayers were assigned to the following treatments: (i) 10 ng/mL IL-1B, (ii) 100 nM 666-15 [CREB inhibitor (Li et al., 2016)], (iii) 666-15 plus IL-1B, or (iv) vehicle control. Six different wells (replicates) per treatment were studied. To mimic the *in vivo* study conditions, the medium was renewed every 24 h with above treatments for four consecutive days. After 4 days of treatment, cells were harvested with QIAzol (Qiagen, Hilden, Germany), snap frozen and stored at −80°C until RNA isolation. The dose of 666-15 used in this study is below the dose that elicits off-target effects [off-target effects observed at >2 μM *in vivo* (Li et al., 2016)].
B) IL-1B treatment and cell cycle experiment. Cells (passage 105) were seeded onto 12-well plates. At subconfluency (90%), monolayers were assigned to the following treatments: (i) 10 ng/mL IL-1B or (ii) vehicle control. Three different wells (replicates) per treatment were studied. The medium was renewed every 24 h with above treatments for four consecutive days. After 4 days of treatment, cells were harvested with QIAzol (Qiagen, Hilden, Germany), snap frozen and stored at −80°C until RNA isolation.
C) IL-1B treatment experiments to assess cAMP levels. H322 cells were seeded at 10,000 cells per well in a 96-well plate with (i) 10 ng/mL IL-1B (*n* = 7) or (ii) vehicle control (*n* = 8) for 8 h. Two different wells (replicates) per treatment were studied. Details about cAMP assay are in a section below.
D) H322 cells treated with recombinant human CREB protein. H322 cells were seeded at 250,000 cells per well in a 24-well plate. Human recombinant CREB protein Using data provided by Novus Biologics website, we estimated that 10,000,000 cells contain approximately 10 ng of CREB. Therefore, to double the approximated amount of CREB available to a cell, we treated ∼250,000 cells with 250 picograms of human recombinant CREB (in addition to the ∼250 picograms of endogenous CREB). To do this, CREB (H00001385; Novus Biologicals) was resuspended in complete media. Vehicle control was 50 mM Tris-HCl (pH 8.0) diluted in complete media to a final concentration of 1.25 mM Tris-HCl (to match the final concentration of Tris-HCl in the recombinant CREB treatments group). The medium was renewed every 24 h with above treatments for four consecutive days. After 4 days of treatment, cells were harvested with QIAzol (Qiagen, Hilden, Germany), snap frozen and stored at −80°C until RNA isolation.

### Measurement of cAMP

The cAMP-Glo Max Assay (Promega, cat. #V1681) was followed according to the manufacturer’s instructions. Briefly, H322 cells were seeded at a density of 10,000 cells/well in 96-well clear bottom assay plate (Corning, cat. #3610). The next day, cells were stimulated with 10 ng/mL IL-1B (*n* = 7) or vehicle control (*n* = 8) for 8 h in serum-free media according to the manufacturer’s instructions. An 8 h time point was chosen based upon data in human myometrial cells showing that IL-1B increases cAMP levels through a prostaglandin E2 dependent pathway after 5 h of IL-1B treatment that peaks at 12 h (Oger et al., 2002). A standard curve was constructed according to the manufacturer’s instructions. The plate was read on an Agilent Bio Tek Syngery LX multi-mode reader using endpoint luminescence protocol. The cAMP concentrations for vehicle and IL-1B-treated H322 cells were calculated using the values from the standard curve and a sigmoidal 4PL curve (GraphPad Prism 9).

### RNA isolation and qRT-PCR

RNA from the cranial right lung lobe and human NCI-H322 cells was isolated using RNeasy Lipid Tissue kit (Qiagen) with in-column DNase digestion (Qiagen). RNA concentration was measured with a NanoDrop (ThermoFisher Scientific). Total RNA (2,000 ng) was reverse transcribed using Superscript VILO Master Mix (ThermoFisher Scientific). Transcripts for *Muc5ac*, *Muc5b*, *purinergic receptor P2Y2,* (*P2ry2*)*, Muc1, Muc4, cholinergic receptor muscarinic 3 (M3R)* and *Il1r1* were quantified in mouse lung homogenates using primers designed in PrimerQuest (IDT; idtdna.com) and based on *Mus musculus* mRNA GenBank (NCBI; www.ncbi.nlm.nih.gov) sequences. *Muc5ac*, *Muc5b*, *purinergic receptor P2Y2,* (*P2ry2*)*, Muc1, Muc4, cholinergic receptor muscarinic 3* (*M3R*) were quantified in lung samples from mice that did not undergo flexiVent. Transcripts for *CREB1, BDNF, SPDEF*, *FOXA2* and *ILIR1* were quantified in NCI-H322 cells using primers based on *Homo sapiens* mRNA GenBank sequences. All primers are listed is Supplementary Table S1. PCR reactions were carried out in 96-well plates with fast SYBR Green master mix (Applied Biosystems, Waltham, MA, US). PCR parameters included denaturation at 95°C for 10 min, followed by 45 cycles of 10 s at 95°C, annealing at 60°C for 10 s, and extension at 72°C for 10 s. A melting curve was performed at the end to confirm presence of single amplicons. Primer pair specificity was further confirmed by electrophoresis of single band PCR products. Relative abundances were calculated using the 2^−ΔΔCT^ method (Livak and Schmittgen, 2001). Actin beta (*Actb*) was used as endogenous control for mouse lung samples, and ribosomal protein L13a (*RPL13A*) for human cells. Statistical analysis confirmed that there was no influence of treatment or genotype on *Actb* expression nor was there an influence of treatment on *RPL13A* expression.

### End-point PCR for *Creb1* mRNA

PCR reactions were performed using Platinum PCR SuperMix High Fidelity (Invitrogen) and included an initial denaturation at 94°C for 30 s, followed by 45 cycles of 15 s at 94°C, annealing at 56°C for 30 s, and extension at 68°C for 1 min. A no template control reaction (water replacing template cDNA) was included. The *Creb1* ^*fl/fl*^ mouse line used in the current study has exon 2 of the *Creb1* gene flanked with loxP sites; therefore, Cre-mediated recombination results in excision of the exon 2 (Covington et al., 2011). To assess recombination, primers spanning exons 1 to 4 of *Creb1* were used in lung homogenates to detect the wild-type and truncated *Creb1* mRNA fragments. For more details, please refer to Supplementary Figure S2 in this manuscript. Primer sequences are shown in Supplementary Table S1. PCR products were electrophoresed in a 1.8% agarose gel and gel-purified DNA fragments were submitted for sequencing to confirm excision of exon 2.

### Inflammatory-directed arrays

Lung cDNA samples were analyzed by the mouse innate and adaptive immune responses RT^2^ profiler PCR array (PAMM-052ZA; Qiagen) in a subset of mice (*n* = 6 per group). Real time qPCR data for 84 target genes (Supplementary Table S2) were acquired using fast SYBR Green master mix (Applied Biosystems) and a LightCycler 96 (Roche). PCR included denaturation at 95°C for 10 min, followed by 50 cycles of 10 s at 95°C, annealing at 60°C for 10 s, and extension at 72°C for 10 s. Melting curves were performed. Relative abundances were calculated using the 2^−ΔΔCT^ method (Livak and Schmittgen, 2001). *Actb*, *B2m*, *Gapdh*, *Gusb* and *Hsp90ab1* were endogenous controls.

### Cell cycle-directed arrays

cDNA from vehicle-treated (*n* = 3) and IL-1B treated H322 cells (*n* = 3) were analyzed by the human cell cycle RT^2^ profiler PCR array (PAHS-020Z; Qiagen). Real time qPCR data for 84 target genes (Supplementary Table S3) were acquired using fast SYBR Green master mix (Applied Biosystems) and a LightCycler 96 (Roche). PCR included denaturation at 95°C for 10 min, followed by 50 cycles of 10 s at 95°C, annealing at 60°C for 10 s, and extension at 72°C for 10 s. Melting curves were performed. Relative abundances were calculated using the 2^−ΔΔCT^ method (Livak and Schmittgen, 2001). *ACTB*, *B2M*, *GAPDH*, *HPRT1* and *RPLP0* were endogenous controls.

### Chromatin immunoprecipitation (ChIP)

NCI-H322 cells (8 × 10^6^) at confluency were stimulated with 10 µM forskolin (Millipore Sigma) for 15 min to activate CREB signaling (Seternes et al., 1999). Cells were crosslinked in 1% formaldehyde/growth medium for 10 min at 37°C and then washed twice with ice-cold DPBS (Ca^2+^/Mg^2+^ free). ChIP assay was performed using the EZ-ChIP kit (17-295; Millipore Sigma) according to the manufacturer’s instructions. Briefly, cells were harvested and resuspended in SDS lysis buffer with proteinase inhibitors (A32953, ThermoFisher Scientific) and equally divided into two microtubes. After a 10-min incubation on ice, cell lysates were sonicated using a 2800 Branson (Branson Ultrasonics, Danbury, US) ultrasonic water bath (25 cycles of 40 kHz for 30 s with 30 s rest intervals in ice water). The sonicated chromatin was clarified by centrifugation at 15,000 x g for 10 min at 4°C. Supernatants were then diluted in ChIP dilution buffer and pre-cleared with Protein A agarose/Salmon Sperm DNA 50% slurry for 30 min at 4°C. Immunoprecipitation was performed overnight at 4°C with rabbit anti-phospho-CREB (1:100 dilution; 9198S; Cell Signaling, Danvers, MA, US) antibody or mouse normal IgG as a control. Immune complexes were captured with Protein A agarose/Salmon Sperm DNA 50% slurry for 2 h at 4°C. Beads were spun at 1,000 x g for 1 min and washed in low salt buffer, high salt buffer, LiCl buffer, and twice with TE buffer. Protein-DNA complexes were eluted from beads twice by addition of elution buffer (1% SDS, 0.1 M NaHCO_3_). Beads were collected by centrifugation at 1,000 x g for 1 min and supernatant transferred to a new tube. Eluates were pooled, added NaCl (0.2 M) and incubated at 65°C for 4 h to reverse crosslinking. Samples were then treated with Proteinase K at 45°C for 1 h and purified using a Qiaex II gel extraction kit (Qiagen).

### End-point PCR for ChIP DNA fragments

PCR was performed using Platinum PCR SuperMix High Fidelity (Invitrogen). PCR parameters included an initial denaturation at 94°C for 30 s, followed by 40 cycles of 15 s at 94°C, annealing at 56.5°C–57°C for 30 s, and extension at 68°C for 30 s. A no template control reaction (water replacing DNA) was included. Human genomic DNA was used as positive control. Primers for *FOXA2* and *SPDEF* were designed from regions containing a cAMP response element (CRE) site in the promoter, which CREB binds to. For *Homo sapiens SPDEF* (NM_012391.3), a ∼4 kb region upstream to the transcriptional start site was scanned, and a half CRE site [CGTCA (Mayr and Montminy, 2001)] was found at ∼3.2 kb upstream to the transcriptional start site. A half CRE site (CGTCA) was found at ∼180 bp upstream to the transcriptional start site of the *Homo sapiens FOXA2* (NM_021784.5). Human genomic DNA isolated from NCI-H322 cells was used as positive control for the PCR reactions. Primers targeting the promoter region of a non-CREB-regulated gene (*GAPDH*) were used as a negative control. All primers are listed in Supplementary Table S1. PCR products were electrophoresed in a 1.5% agarose gel.

### Chemicals and drugs

Acetyl-beta-methacholine-chloride (Sigma) was dissolved in 0.9% saline for flexiVent studies. Mouse IL-1B (R&D Systems, MN, US) was dissolved in 0.9% sterile saline containing 0.1% BSA carrier to a concentration of 10 mg/mL, aliquoted and stored at −20°C until use. A new aliquot of mouse IL-1B (10 mg/mL) stock was diluted 1:1,000 into 0.9% sterile saline for intranasal delivery each day. Recombinant human IL-1B (R&D Systems) was dissolved in PBS to a concentration of 100 mg/mL, aliquoted and stored at −20°C until use. A new aliquot of human IL-1B (100 mg/mL) stock was diluted 1:10,000 into complete growth media for cell culture experiments each day. The CREB inhibitor 666-15 (Li et al., 2016) (R&D Systems) was initially dissolved in 100% DMSO at a concentration of 1 mM. The stock was diluted 1:10,000 into complete growth media for cell culture experiments.

### Statistical analysis

A three-way ANOVA was performed for flexiVent studies with methacholine dose as a repeated measure, and genotype and treatment as main factors. A three-way ANOVA was performed for array studies with gene, genotype, and treatment as factors. A three-way ANOVA was performed for goblet cell analysis, with genotype, treatment, and methacholine as main factors. A two-way ANOVA was performed for all other analyses with genotype and treatment as main factors. Significance for main effects and interactions was set at *p* < 0.05. Post hoc comparisons were performed using a Sidak’s multiple comparisons test; major comparisons of interest were: 1) Creb1^fl/fl^Scgb1a1^wt^ + IL-1B vs. Creb1^fl/fl^Scgb1a1^wt^ + vehicle; and 2) Creb1^fl/fl^Scgb1a1^cre^ + IL-1B vs. Creb1^fl/fl^Scgb1a1^cre^ + vehicle. Similarly, for human cell studies, a two-way ANOVA was performed with CREB inhibitor and IL-1B treatment as main factors. Significance for main effects and interactions was set at *p* < 0.05. For CREB overexpression studies and cAMP assay, a student’s unpaired *t*-test was performed with significance set at *p* < 0.05. Post hoc comparisons for human studies were performed using a Sidak’s multiple comparisons test; major comparisons of interest were: 1) vehicle + vehicle vs. IL-1B + vehicle; and 2) CREB inhibitor + vehicle vs. CREB inhibitor + IL-1B. For inflammatory-directed PCR array data, an FDR of <0.05 was incorporated. Statistical analyses were performed using GraphPad Prism 9.0a. Data are presented as mean ± SEM.

## Results

### Repeated IL-1B administration increases the percentage of club cells expressing Creb1 in murine airways

We first confirmed that our tamoxifen dosing and duration induced Cre-recombinase expression in Creb1^fl/fl^Scgb1a1^cre^ mice. Transcripts for *Cre* were detected in Creb1^fl/fl^Scgb1a1^cre^ lung homogenates, with Ct values averaging 21.46 ± 0.28, whereas only background levels were detected in the Creb1^fl/fl^Scgb1a1^wt^ samples. For reference, Ct values for *actin* in the same Creb1^fl/fl^Scgb1a1^cre^ lung homogenates averaged 15.11 ± 0.17. We directly confirmed recombination in Creb1^fl/fl^Scgb1a1^cre^ mice by probing reverse transcribed mRNA in lung homogenates with end-point PCR. A 436 bp band that corresponded to the wild-type *Creb1* transcript was observed in both Creb1^fl/fl^Scgb1a1^wt^ and Creb1^fl/fl^Scgb1a1^cre^ lung homogenates (Supplementary Figure S2). Creb1^fl/fl^Scgb1a1^cre^ mice also displayed a shorter 314 bp-fragment that corresponded to the truncated *Creb1* mRNA due to excision of exon 2 (Covington et al., 2011). Two bands were expected in Creb1^fl/fl^Scgb1a1^cre^ mice given that the lung homogenate contains mixed populations of cells. Sanger sequencing of gel-purified PCR products confirmed excision of exon 2 in the shorter fragment, as well as full length wild type *Creb1* (Supplementary Figure S2).

To assess recombination rates, we utilized ROSA^mT/mG^ Creb1^fl/fl^Scgb1a1^cre^ mice. These mice have cell membrane tdTomato expressed that upon recombination, is replaced with membrane localized GFP (Supplementary Figure S3A). We measured the percentage of CCSP-labeled cells that expressed GFP in a small subset of mice (Supplementary Figure S3B). This analysis demonstrated that approximately 75% of the CCSP-labeled cells that we assessed expressed GFP (Supplementary Figure S3C). This finding is consistent with the original study publishing the parent *Scgb1a1* ^tm1(cre/ERT)Blh^ mouse line, in which two tamoxifen injections were sufficient to induce Cre expression in 50%–60% of bronchiolar club cells (Rawlins et al., 2009).

**FIGURE 1:**
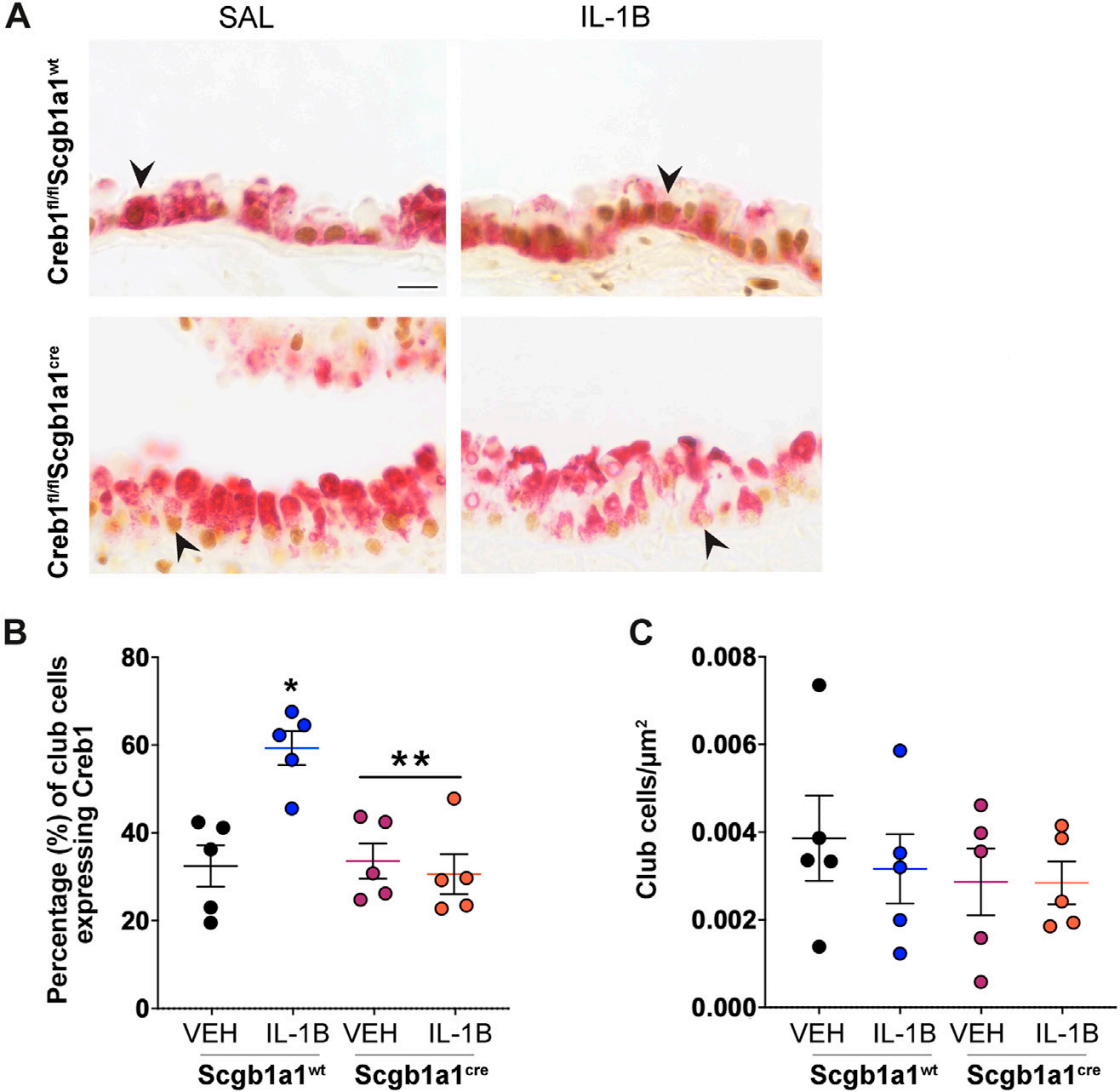
Percentage of club cells expressing Creb1 is increased following repeated administration of IL-1B. **(A)** Representative images of dual label immunohistochemistry for Creb1 (brown) and clara cell secretory protein (red) in airway cross sections from Creb1^fl/fl^Scgb1a1^cre^ or Creb1^fl/fl^Scgb1a1^wt^ mice. Arrow indicates an example of CCSP-positive (club) cell expressing presumably nuclear staining for Creb1. Scale bar is 20 μm. **(B)** Percentage of club cells expressing Creb1. * = compared to vehicle control, *p* = 0.0013; ** = main genotype effect across treatments, *p* = 0.0055. **(C)** Density of club cells in airway. For B and C, individual points are data collected from a single mouse. n = 5 per group. Abbreviations: WT, wild type; Scgb1a1^cre^, club cell promoter driving CRE recombinase; IL-1B, Interleukin 1β; VEH, vehicle.

We next examined the impact of IL-1B administration on Creb1 induction in the airways. Using double label immunohistochemistry for Creb1 and the club cell marker SCBA1A1, which is also known as CCSP (Martinu et al., 2023) and what we refer to hereafter, we found that IL-1B administration in wild type mice increased the percentage of club cells expressing Creb1 compared to vehicle control (Figures 1A, B). As expected, conditional elimination of Creb1 in club cells prevented IL-1B-mediated induction of Creb1 (Figure 1A). Approximately 30% of the club cells in the Creb1^fl/fl^Scgb1a1^cre^ mice expressed Creb1, which is consistent with Supplementary Figure S3C demonstrating that only ∼75% of the club cells express *Cre*. Therefore, we expect that ∼25% of the club cells from Creb1^fl/fl^Scgb1a1^cre^ mice would still express Creb1 because they lack *Cre*. Expression of Creb1 in club cells of Creb1^fl/fl^Scgb1a1^wt^ mice might represent basal Creb1 expression or expression due to aspiration of saline vehicle control. No differences in the density of club cells were noted across treatments or genotypes (Figure 1C). These results demonstrated that IL-1B increased Creb1 in club cells.

### IL-1B increases mRNA expression of secreted and tethered mucins

**FIGURE 2:**
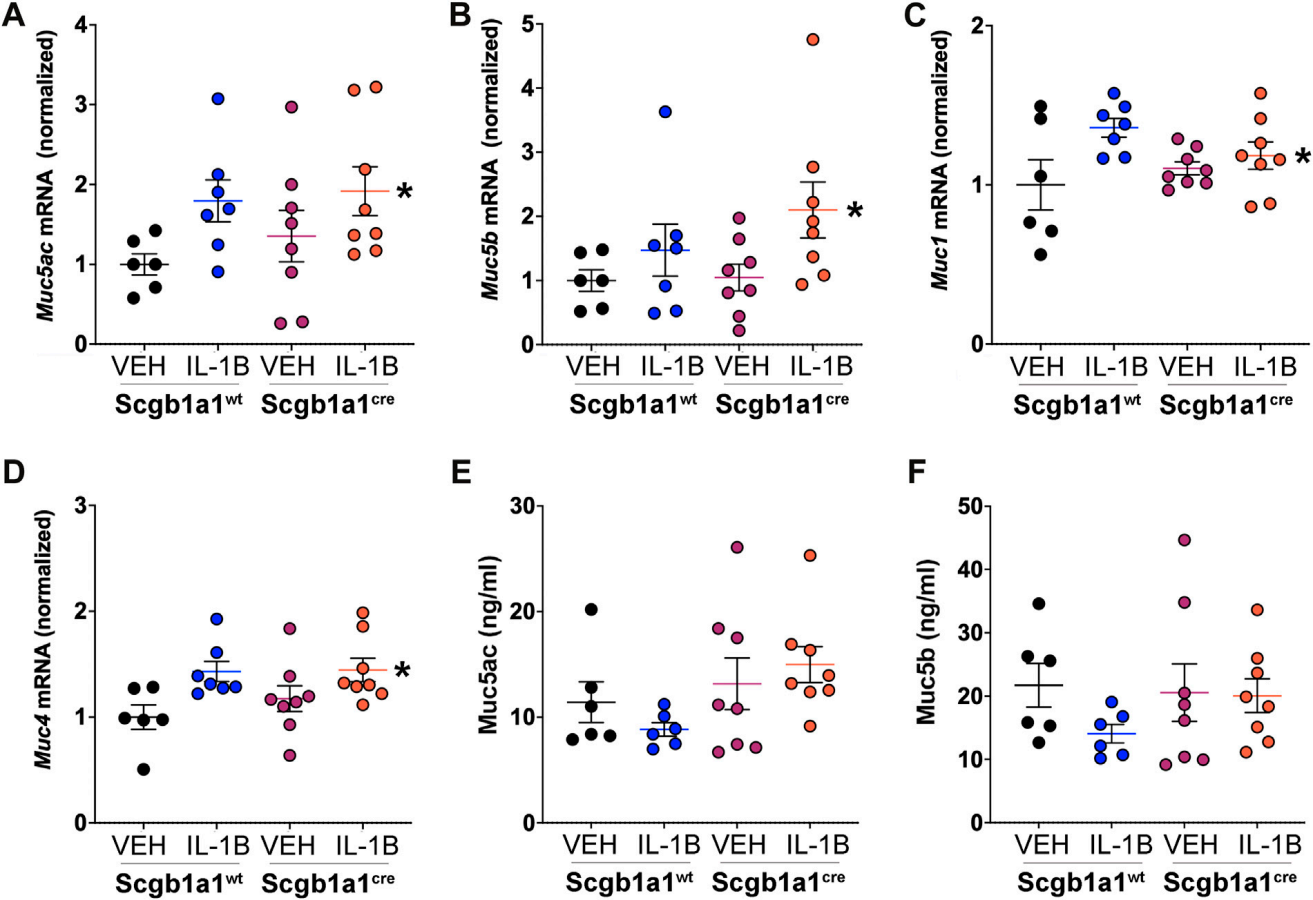
IL-1B treatment increases mRNA abundance of secreted and tethered mucins. Abundance of mRNA in the murine lung for **(A)** *Muc5ac,* * = main effect of IL-1B treatment across genotype, *p* = 0.0246; **(B)** *Muc5b,* * = main effect of IL-1B treatment across genotype, *p* = 0.0342; **(C)** *Muc1,* * = main effect of IL-1B treatment across genotype, *p* = 0.0206; and **(D)** *Muc4* * = main effect of IL-1B treatment across genotype, *p* = 0.0048. Bronchoalveolar lavage concentrations of **(E)** Muc5ac and **(F)** Muc5B. A trend for statistical significance for main genotype effect across treatment was observed for Muc5ac concentrations (*p* = 0.054). For all panels, individual points are data collected from a single mouse: Creb1^fl/fl^Scgb1a1^wt^ mice treated with vehicle (*n* = 6) or IL-1B (*n* = 7); Creb1^fl/fl^Scgb1a1^cre^ mice treated with vehicle (*n* = 8) or IL-1B (*n* = 8). For panel E and F, a single mouse from the Creb1^fl/fl^Scgb1a1^cre^ treated with vehicle group was a statistical outlier using Grubbs outlier test. Therefore, concentrations of Muc5ac and Muc5b from this mouse were not included. Abbreviations: WT, wild type; Scgb1a1^cre^, club cell promoter driving CRE recombinase; IL-1B, Interleukin 1β; VEH, vehicle.

Prior studies have demonstrated that IL-1B is associated with increased expression of human *MUC5AC* and *MUC5B* mRNA (Chen et al., 2019) and protein levels (Song et al., 2003; Fujisawa et al., 2011). Assessment of *Muc5ac* and *Muc5b* in murine lung homogenates revealed main effects of IL-1B treatment (Figures 2A, B). *Muc1* is a membrane mucin that acts as an adhesion site for *Pseudomonas aeruginosa* in the airway (Lillehoj et al., 2001). Prior studies in oral epithelial cells indicated that IL-1B treatment increases *Muc1* mRNA (Li et al., 2003). Therefore, we also examined *Muc1* expression in the airways and found a main treatment effect of IL-1B (Figure 2C). Similarly, *Muc4*, another membrane bound mucin in the airway that is regulated by neutrophil elastase (Fischer et al., 2003), was also elevated by IL-1B treatment in both wild type mice and mice with conditional loss of club cell Creb1 (Figure 2D). These studies were consistent with prior work indicating that IL-1B regulates transcription of secreted and tethered mucins.

To determine if parallel increases in mucin proteins were observed, we measured concentrations of the secreted mucins, Muc5ac and Muc5b, in the bronchioalveolar lavage fluid. We did not measure Muc1 and Muc4 in the bronchoalveolar lavage fluid since they are membrane bound. Muc5ac and Muc5b protein concentrations in response to IL-1B did not parallel the increased Muc5ac and Muc5B mRNA expression (Figures 2E, F). Instead, we found a trend (*p* = 0.054) for conditional loss of club cell Creb1 to increase the concentrations of Muc5ac in the bronchoalveolar lavage fluid (Figure 2E). Muc5b bronchoalveolar lavage fluid concentrations did not differ statistically across treatment or genotype groups (Figure 2F).

### Loss of club cell Creb1 modifies mucin secretion machinery

**FIGURE 3:**
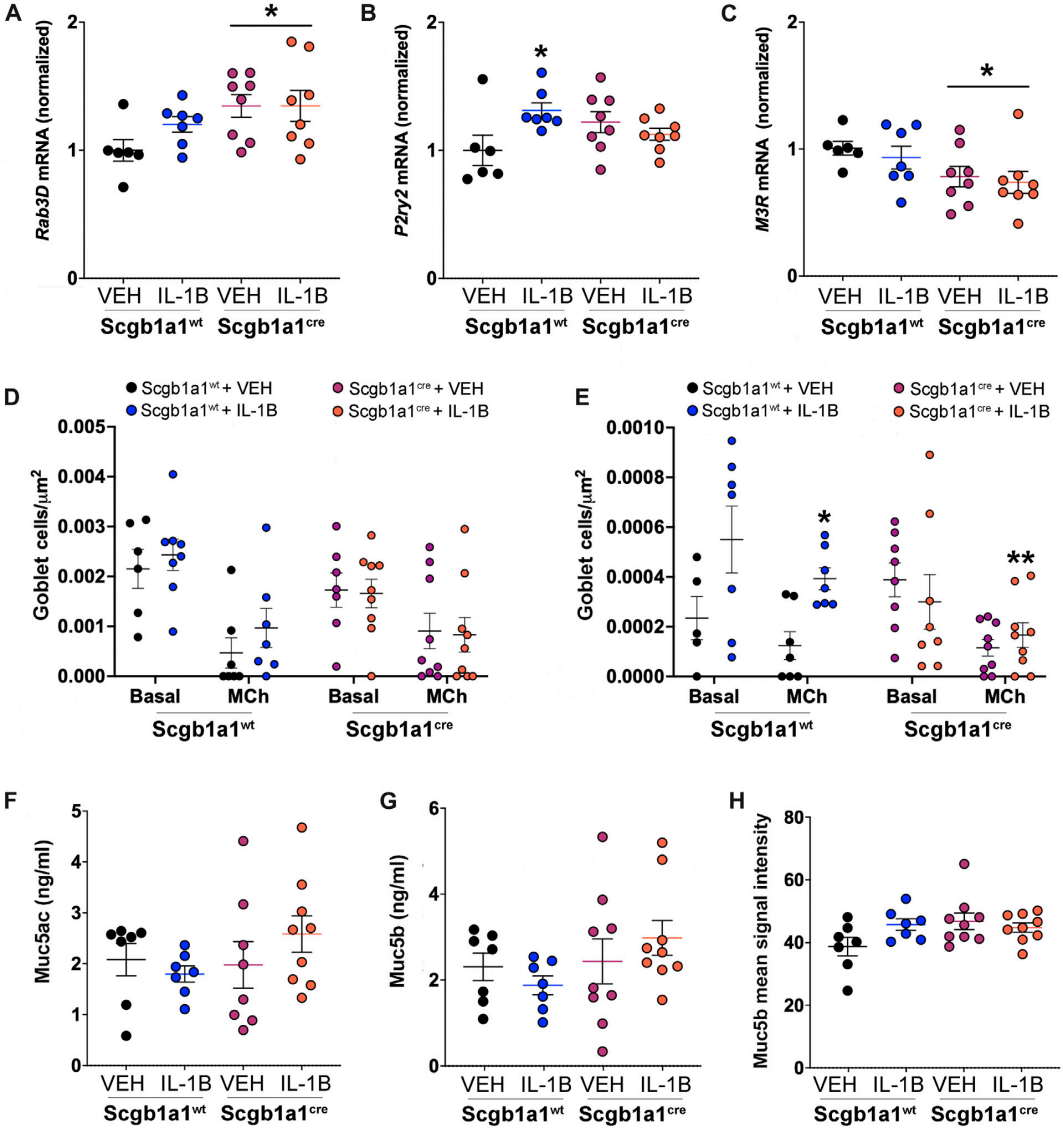
Conditional loss of club cell Creb1 modifies mucin secretion characteristics. **(A)** Abundance of mRNA in the murine lung for **(A)** *Rab3D,* * = main effect of genotype across treatment, *p* = 0.0280; **(B)** *P2ry2,* * = compared to vehicle control, *p* = 0.0233; Significant genotype x treatment interaction, *p* = 0.0142; **(C)** *M3R,* * = main effect of genotype across treatment, *p* = 0.0179; and **(D)** *Muc4,* * = main effect of IL-1B treatment across genotype, *p* = 0. 0048. **(D)** Density of goblet cells staining for Alcian-Blue/PAS under basal and methacholine stimulated conditions. Density is for cells with acidic mucins, as detected by dark blue staining. Main effect of methacholine, *p* < 0.0001. **(E)** Density of goblet cells staining for Alcian-Blue/PAS under basal and methacholine stimulated conditions. Density is for cells with neutral mucins, as detected by purple/magenta staining. Main effect of methacholine, *p* = 0.0036. Significant treatment x genotype interaction, *p* = 0.0069. * = compared to Creb1^fl/fl^Scgb1a1^wt^ vehicle control under methacholine stimulated conditions, *p* = 0.0028; ** = to Creb1^fl/fl^Scgb1a1^wt^ IL-1B under methacholine stimulated conditions, *p* = 0.0067. Post methacholine bronchoalveolar lavage concentrations of **(F)** Muc5ac and **(G)** Muc5b. **(H)** Mean immunohistochemistry signal intensity of Muc5b in the airway post methacholine stimulation. For all panels, individual points are data collected from a single mouse. For Panel F, one mouse from the Creb1^fl/fl^Scgb1a1^cre^ + VEH group had Muc5ac concentrations below detection and was therefore not included in analysis. Abbreviations: WT, wild type; Scgb1a1^cre^, club cell promoter driving CRE recombinase; IL-1B, Interleukin 1β; VEH, vehicle; MCh, methacholine.

We hypothesized that a lack of parallel increases in protein concentrations for Muc5ac and Muc5b could be due treatment and/or genotype dependent modifications in mucin secretion. Therefore, we measured the mRNA expression of three molecules important for mucin secretion (Figure 3). We first measured expression of Rab3D, an exocytic machinery molecule important for vesicle docking that is expressed in club cells (Evans et al., 2004) and goblet cells (Tuvim et al., 2009). We found that loss of club cell Creb1 increased *Rab3D* mRNA in whole lung homogenates (Figure 3A), suggesting that release of mucins may be enhanced (Ohnishi et al., 1997). We also measured the mRNA expression of the P2Y2 purinergic receptor (P2ry2), which regulates mucin secretion under basal conditions in response to ATP (Adler et al., 2013). IL-1B increased *P2ry2* mRNA in the total lung of wild type mice, but not in mice with conditional loss of club cell Creb1 (Figure 3B). Lastly, we measured mRNA expression of the cholinergic muscarinic 3 receptor (M3R), an important regulator of airway mucus secretion and airway smooth muscle contraction (Rogers, 2001). Loss of club cell Creb1 decreased M3R mRNA expression in total lung homogenates (Figure 3C), suggesting that cholinergic mediated secretion may be impaired in mice with loss of club cell Creb1.

### Loss of club cell Creb1 changes the density of goblet cells

Finding that loss of club cell Creb1 modified molecules important for regulated (Rab3D) mucin secretion under basal (P2ry2) and cholinergic stimulated (M3R) conditions suggested that changes in secretion properties might be detected by measuring goblet cell density under basal and stimulated conditions. Thus, we measured goblet cell density using Alcian-Blue/PAS staining in lung sections from mice under basal conditions, and then in a separate group of mice subjected to intra-airway methacholine dose-response curves during airway mechanic (flexiVent) studies. We separated goblet cell density into two groups: cells with acidic mucins, as distinguished with dark blue staining, or cells with neutral mucin, as distinguished with purple staining. As expected, methacholine caused goblet cell discharge, leading significant decreases in detection of goblet cells and subsequent goblet cell density (Figures 3D, E, Supplementary Figures S4A, B). The density of goblet cells containing acidic mucins did not differ across genotype or treatment (Figure 3D). However, the density of goblet cells with neutral mucins was increased in wild type mice treated with IL-1B, but not in mice with conditional loss of club cell Creb1 (Figure 3E, Supplementary Figure S4B).

To further examine secretion, we performed two additional studies. First, we measured BAL concentrations of Muc5ac (Figure 3F) and Muc5b (Figure 3G) from methacholine treated mice that underwent airway mechanic (flexiVent) studies. No statistically significant effects of treatment or genotype were noted. We considered that mucin concentrations in the lavage might provide an incomplete assessment of the amount of mucin in the airways; for example, mucin may adhere to the airway surface and not wash off during the lavage process. This seemed especially relevant given that the BAL concentrations of mucins post methacholine stimulation were less than those found under basal conditions. Therefore, we performed immunohistochemistry on lung sections. We focused on Muc5b since it is the predominant mucin in the mouse airway under basal conditions (Zhu et al., 2008) and is the dominant secreted mucin following chronic IL-1B exposure (Chen et al., 2019). Qualitatively, there was an increase in Muc5b upon IL-1B treatment in wild type mice (Supplementary Figure S4C); however, statistical analysis revealed no main effects of treatment or genotype. A strong trend for a genotype x treatment interaction was noted (*p* = 0.059) (Figure 3H). Combined, these results supported secretion defects and suggested that the release of mucin were impacted by treatment and genotype.

### IL-1B-mediated alterations in airway mechanics are blunted by loss of club cell Creb1 in mice

**FIGURE 4:**
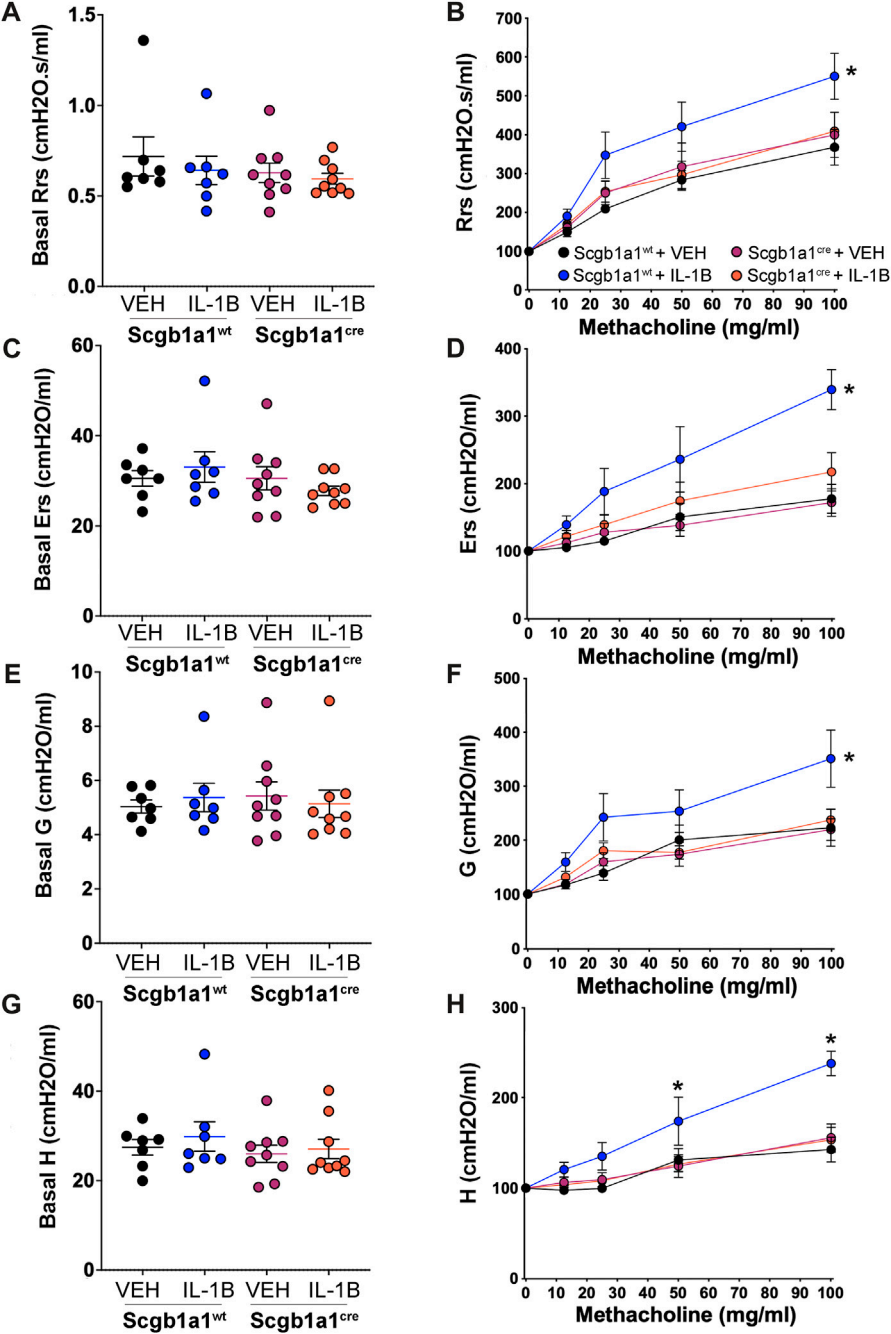
Conditional loss of club cell Creb1 diminishes IL-1B-mediated alterations in pulmonary mechanics. Basal airway resistance **(A)**, airway elastance **(C)**, tissue damping **(E)** and tissue elastance **(G)** as measured with a forced oscillation technique. Methacholine dose response curves normalized to basal values for airway resistance **(B)**, airway elastance **(D)**, tissue damping **(F)** and tissue elastance **(H)**. For Panels **(A, C, E, G)**, individual points are data collected from a single mouse. For Panel **(B)**, * = main effect of IL-1B treatment in wildtype mice compared to wild type vehicle controls, *p* = 0.0005. For Panel **(D)**, * = main effect of IL-1B treatment in wildtype mice compared to wild type vehicle controls, *p* < 0.0001. For Panel **(F)**, * = main effect of IL-1B treatment in wildtype mice compared to wild type vehicle controls, *p* < 0.0001. For Panel **(H)**, a significant genotype x treatment x methacholine dose interaction was noted, allowing for individual *post hoc* comparisons to be conducted. * = IL-1B treatment in wildtype mice compared to wild type vehicle controls; at 50 mg/mL, *p* = 0.0351; at 100 mg/mL, *p* < 0.0001. Legend key in Panel **(B)** applies to Panels **(D, F, H)**. *n* = 7 for Creb1^fl/fl^Scgb1a1^wt^ mice treated with vehicle or IL-1B; *n* = 9 for Creb1^fl/fl^Scgb1a1^cre^ mice treated with vehicle or IL-1B. Abbreviations: WT, wild type; Scgb1a1^cre^, club cell promoter driving CRE recombinase; IL-1B, Interleukin 1β; VEH, vehicle.

Inflammation and mucus accumulation can decrease airway caliber, resulting in higher airway resistance. Consistent with that, humans with CF (Levine et al., 2016) and animal models of CF (Adam et al., 2013) display elevated airway resistance. Using a forced oscillation technique (flexiVent), we measured airway resistance (Figures 4A, B). No differences were noted in basal airway resistance (Figure 4A). However, statistical analysis of normalized airway resistance in response to methacholine, a secretagogue that stimulates mucus secretion and smooth muscle contraction, revealed a significant genotype x treatment interaction. Post hoc comparisons determined that IL-1B increased airway resistance relative to vehicle controls in wild type mice (Figure 4B), but not in mice with loss of club cell Creb1 (Figure 4B).

We also assessed three additional airway mechanic properties that are elevated in murine models of CF (Darrah et al., 2016): airway elastance (Ers, the reciprocal of airway compliance), tissue damping (G, reflects energy dissipation into the alveoli) and tissue elastance (H, reflects energy conservation into the alveoli). No significant genotype (Creb1^fl/fl^Scgb1a1^cre^ vs. Creb1^fl/fl^Scgb1a1^wt^) or treatment (IL-1B vs. VEH) differences were observed in basal airway elastance (Figure 4C), basal tissue damping (Figure 4E) or basal tissue elastance (Figure 4G). However, assessment of normalized responses to the secretagogue and smooth muscle contracting agent methacholine revealed significant genotype x treatment interactions for airway elastance (Figure 4D) and tissue damping (Figure 4F). Post hoc comparisons indicated that IL-1B treatment increased airway elastance (Figure 4D) and tissue damping (Figure 4F) in wild type mice compared to vehicle control, but not in mice with loss of club cell Creb1.

A significant genotype x treatment x methacholine dose interaction was observed for normalized tissue elastance responses (Figure 4H). The three-way interaction allowed *post hoc* comparisons to determine that in wild type mice, IL-1B treatment for 4 days increased elastance at the 50 and 100 mg/mL methacholine concentrations compared to vehicle controls (Figure 4H). This effect was absent in mice with loss of club cell Creb1. These results suggested that IL-1B treatment altered airway mechanics to mimic some features of CF, and that loss of club cell Creb1 mitigated and/or prevented these effects.

### Some pro-inflammatory effects of IL-1B are negated by loss of murine club cell Creb1 post methacholine stimulation

**FIGURE 5:**
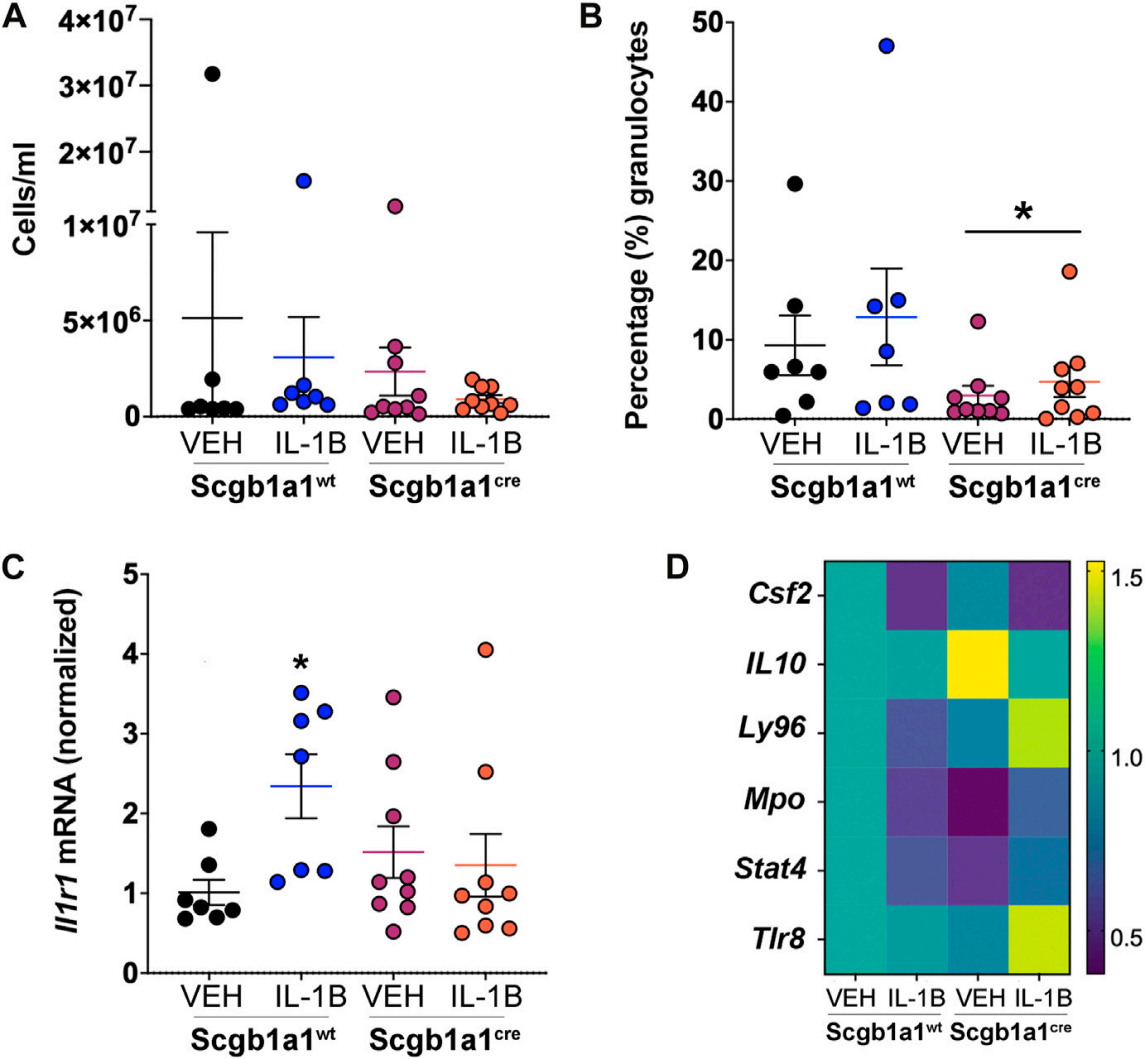
Conditional loss of club cell Creb1 modulates inflammatory responses post methacholine. **(A)** Numbers of cells in the BAL. **(B)** Percentage of cells that were granulocytes in the BAL. * = main effect of genotype across treatments, *p* = 0.0418. **(C)** Expression of lung *Il1r1* mRNA normalized to vehicle control wild type mice. *p = 0.0324. **(D)**. Heat map of genes that were differentially expressed in the murine lung due to treatment and/or genotype. Scale represents fold change. More details are available in Supplementary Table S4. For Panels **(A–C)**, *n* = 7 for Creb1^fl/fl^Scgb1a1^wt^ mice treated with vehicle or IL-1B; *n* = 9 for Creb1^fl/fl^Scgb1a1^cre^ mice treated with vehicle or IL-1B. For Panel **(D)**, *n* = 6 mice per group. Abbreviations: WT, wild type; Scgb1a1^cre^, club cell promoter driving CRE recombinase; IL-1B, Interleukin 1β; VEH, vehicle. *Csf2*, granulocyte macrophage colony stimulating factor; *Il10*, interleukin 10; *Ly96*, myeloid differentiation factor 2; *Mpo*, myeloperoxidase; *Stat4*, signal transducer and activator of transcription 4; *Tlr8*, toll like receptor 8.

We observed conditional loss of club cell Creb1 offered some protection against the IL-1B-mediated pro-mucin phenotypes under basal and methacholine-stimulated conditions, as well as prevented the IL-1B-mediated effects on pulmonary mechanics under methacholine-stimulated conditions. Since M3R receptor expression was decreased by loss of club cell Creb1, and since M3R on neutrophils promote NETosis and can induce secondary cell damage and inflammation (Carmona-Rivera et al., 2017), we considered that inflammation mediated by IL-1B may be reduced post methacholine stimulation. There was no effect of treatment or genotype on the number of cells per ml in the BAL (Figure 5A). However, a main effect of genotype on the percentage of granulocytes in the BAL was noted, with fewer granulocytes (mainly neutrophils) observed in mice with conditional loss of club cell Creb1 (Figure 5B). Since Il1r1 is a marker of neutrophilic inflammation (Evans et al., 2018) and the receptor for IL-1B, we also examined its expression in lung homogenates. We observed a statistically significant genotype x treatment interaction. Post hoc comparisons revealed a significant increase in *Il1r1* mRNA abundance in wild type mice treated with IL-1B compared to vehicle controls, but not in mice with conditional club cell Creb1 loss (Figure 5C).

We further examined inflammatory responses post methacholine stimulation by utilizing innate and adaptive inflammatory-directed PCR arrays (Supplementary Table S2). Statistical analysis revealed significant genotype x treatment interaction. After correcting for false discovery rate, *post hoc* comparisons revealed only 6 genes that were differentially expressed in response to treatment and/or genotype (Figure 5D, Supplementary Table S4). *Ly96* (Lymphocyte Antigen 96), also known as myeloid differentiation factor 2, was the only gene that showed a significant decrease following IL-1B treatment in wild type mice but with an increase following IL-1B treatment in mice with conditional club cell Creb1 loss. In addition to innate and adaptive inflammatory arrays, we also looked at the BAL concentrations of IL-13, an important type II immune mediator that was not captured by the arrays that induces goblet cell metaplasia (Pezzulo et al., 2019). However, only 5 samples (spread across different treatments) had IL-13 concentrations within detectable ranges (data not shown). Collectively, these data suggested that conditional loss of club cell Creb1 selectively modulated the proinflammatory actions of IL-1B *in vivo* in whole mouse lungs post methacholine stimulation.

### Genes necessary for human goblet cell expansion are regulated by CREB *in vitro*

The data generated from our *in vivo* mouse studies suggested an important role for club cell Creb1 in regulating the proinflammatory and pro-mucin activities of IL-1B. However, the cellular complexity of the murine lung and whole-body nature of our *in vivo* studies limited our ability to gain mechanistic insights into the potential pathways contributing to the effect observed by loss of club cell Creb1. Therefore, we adopted a reductionist approach and studied a human cell line (NCI-H322) with characteristics of club cells (Lau et al., 1987; Schuller et al., 1991). To mimic *in vivo* mouse conditions, we treated NCI-H322 cells with IL-1B or vehicle control for four consecutive days. Treatment groups were further stratified to receive the CREB inhibitor 666-15 or vehicle control.

**FIGURE 6:**
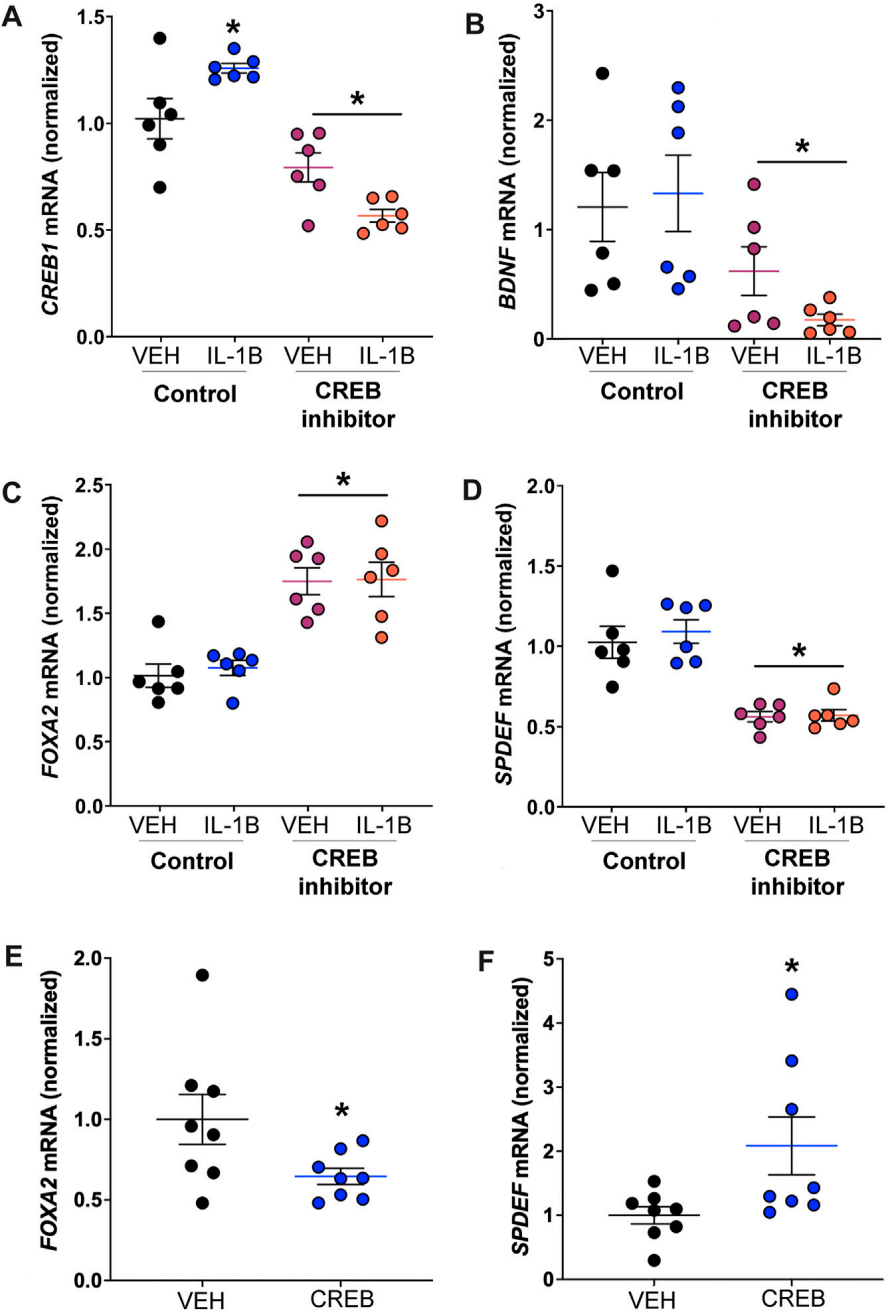
CREB regulates transcripts necessary for goblet cell expansion in human cell line reportedly consisting of club cells. **(A)** Abundance of *CREB1* mRNA in response to IL-1B and/or CREB inhibitor. * = compared to respective vehicle-treated cells, *p* < 0.05. **(B)** Abundance of *BDNF* mRNA; * = main effect of CREB inhibition across treatment, *p* = 0.0033. **(C)** Abundance of *FOXA2* mRNA; * = main effect of CREB inhibition across treatment, *p* < 0.0001. **(D)** Abundance of *SPDEF* mRNA; * = main effect of CREB inhibition across treatment, *p* < 0.0001. **(E)** Abundance of *FOXA2* mRNA in cells treated with recombinant CREB protein; * = compared to vehicle control, *p* = 0.0480. **(F)** Abundance of *SPDEF* mRNA in cells treated with recombinant CREB protein; * = compared to vehicle control, *p* = 0.0371. Abbreviations: IL-1B, Interleukin 1β; VEH, vehicle; *CREB1*, cAMP responsive element binding protein 1; *BDNF*, brain derived neurotrophic factor; *FOXA2*, forkhead box A2; *SPDEF*, SAM pointed domain containing ETS transcription factor.

We first examined *CREB1* mRNA to corroborate our *in vivo* findings that IL-1B treatment increased expression of club cell Creb1 in mice. We found a significant treatment x drug interaction, with *post hoc* analysis indicating that IL-1B treatment increased *CREB1* mRNA abundance relative to vehicle control (Figure 6A). Pharmacologic CREB inhibition in NCI-H322 cells prevented the IL-1B-mediated increases in *CREB1* mRNA (Figure 6A). As a confirmation of effective CREB inhibition, we also examined the abundance of transcripts for *brain derived neurotrophic factor* (*BDNF*), a CREB target gene (Tao et al., 1998). Pharmacologic inhibition of CREB significantly reduced *BDNF* mRNA (Figure 6B).

Goblet cell expansion is regulated by several key transcription factors. One of these is the protein FOXA2, which is known to repress mucin gene expression and goblet cell expansion (Wan et al., 2004). One study suggested that CREB directly regulates *FOXA2* expression in a mouse pancreatic cell line (Zhang et al., 2005). Therefore, we examined FOXA2 and discovered that pharmacologic CREB inhibition significantly increased *FOXA2* mRNA (Figure 6C). Additionally, prior work suggested that FOXA2 inhibits *SAM pointed domain containing ETS transcription factor (SPDEF)* (Chen et al., 2010), a transcription that drives goblet cell expansion (Kim et al., 2019). Therefore, since CREB inhibition increased *FOXA2* expression, we predicted that *SPDEF* mRNA abundance would be reduced in response to CREB inhibition. Consistent with this, a main effect for CREB inhibition to reduce *SPDEF* transcript abundance was noted (Figure 6D).

We also measured FOXA2 and SPDEF mRNA in H322 cells treated with recombinant CREB. Recombinant CREB caused an approximately 35% reduction in the mRNA expression of FOXA2 in H322 cells (Figure 6E). Conversely, recombinant CREB increased mRNA expression of SPDEF in H322 cells (Figure 6F). These data supported that CREB regulated transcription factors important for mucin synthesis and goblet cell expansion.

**FIGURE 7:**
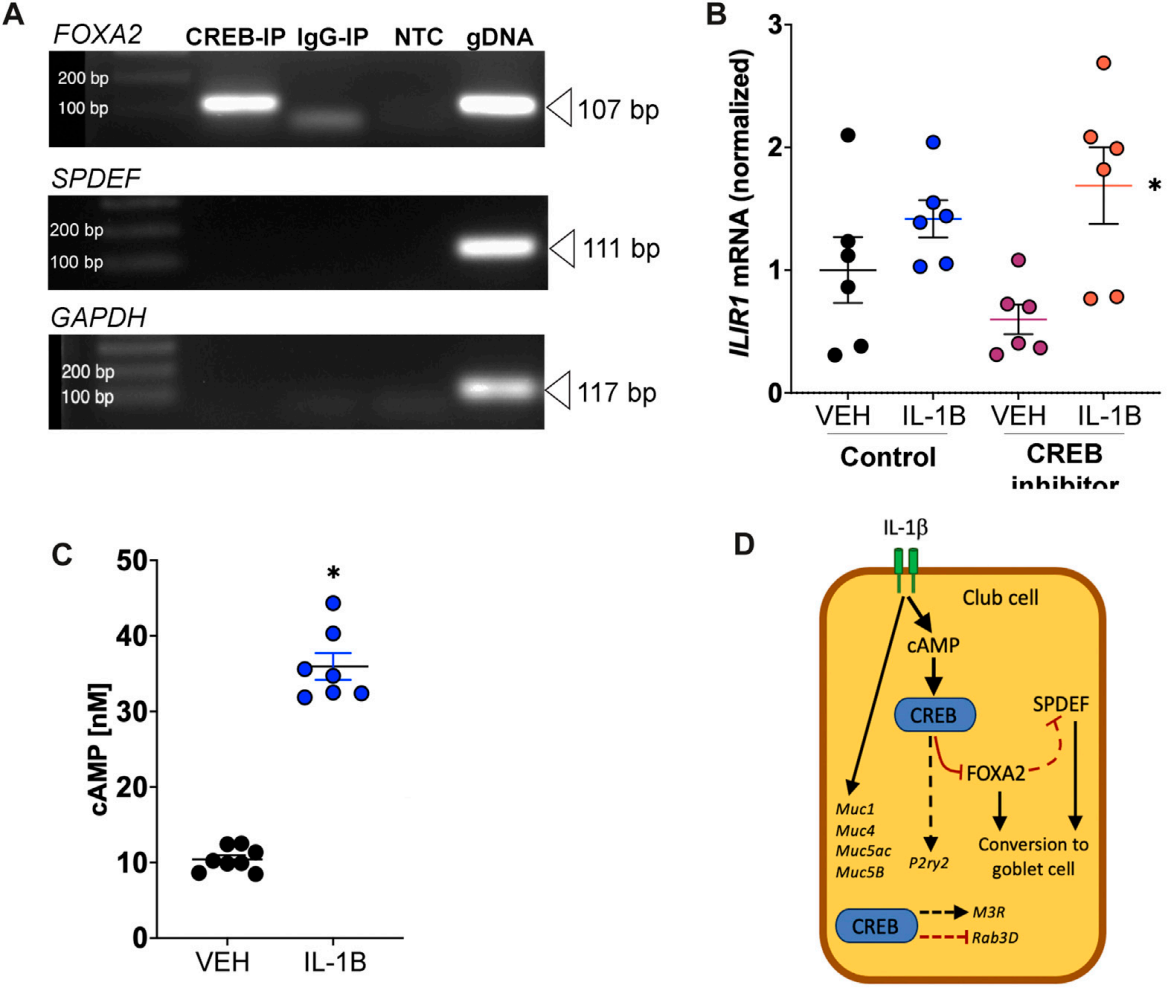
IL-1B increases cAMP concentrations and CREB directly interacts with FOXA2. **(A)** Forskolin-treated NCI-H322 cells were subjected to ChIP assays using primers designed encompassing CRE sites (binding site for CREB). End-point PCR was used to assess template DNA amplification in CREB or IgG immunoprecipitated samples. Genomic DNA was used as positive control for the PCR reactions. **(B)** Abundance of *IL1R1* mRNA in H322 cells treated with IL-1B and/or CREB inhibitor. * = main effect of IL-1B treatment across groups, *p* = 0.0035. **(C)** Concentrations of cyclic AMP (cAMP) in H322 cells treated with IL-1B for 8 h. * = compared to vehicle control, *p* < 0.0001. **(D)** Proposed mechanism of club cell CREB-dependent pathways contributing to mucin prosecretory effects of IL-1B. Dashed line represents hypothetical relationship that is supported by our data and the literature. IL-1B did not modify M3R and Rab3D, but loss of CREB did. Abbreviations: IL-1B, Interleukin 1β; VEH, vehicle; *CREB1*, cAMP responsive element binding protein 1; *FOXA2*, forkhead box A2; *SPDEF*, SAM pointed domain containing ETS transcription factor; *GAPDH*, glyceraldehyde-3-phosphate dehydrogenase; IP, immunoprecipitated; NTC, no template control; gDNA, genomic DNA; *Muc5ac*, Mucin5ac; *Muc5b*, Mucin5b; *Muc1*, Mucin1; *Muc4*, Mucin4; *Rab3D,* RAS oncogene famil*y; M3R*, muscarinic receptor 3.

To understand whether CREB regulation of *FOXA2* or *SPDEF* in the NCI-H322 cell line was via direct interactions, we performed ChIP assays. We discovered that CREB directly bound the *FOXA2* promoter, suggesting direct regulation of *FOXA2* (Figure 7A). Conversely, we found no evidence that CREB directly bound to the promoter region of *SPDEF* (Figure 7A). For these studies, we tested four different sets of primers covering the predicted cAMP response element that binds CREB and none yielded bands after end-point PCR.

To mechanistically link IL-1B to CREB signaling, we performed two experiments. First, we measured IL1R1 mRNA in H322 cells. The average cycle threshold (Ct) for IL1R1 mRNA expression in H322 cells was 23.12 ± 0.14 (SEM), *n* = 6. Like what we found in the lung homogenates of mice treated with IL-1B (Figure 5C), treatment of H322 cells with IL-1B increased IL1R1 mRNA expression (Figure 7B). Pharmacologic CREB inhibition did not prevent IL-1B mediated increase in IL1R1 mRNA expression. Second, we also measured cAMP levels in H322 cells post IL-1B stimulation. We found that IL-1B lead to robust increases in cAMP concentration 8 h after stimulation (Figure 7C). These findings were consistent with work showing a similar increase in cAMP concentrations in response to IL-1B in human myometrial cells that peaked after 12 h of stimulation (Oger et al., 2002) and work showing IL-1B increases cAMP in human airway epithelia (Gray et al., 2004; Clayton et al., 2005).

Lastly, since club cell to goblet cell transition does not involve mitosis, we also examined whether IL-1B modified the mitotic rate of H322 cells using cell-cycle direct arrays (Supplementary Table S3). We found no main effect of treatment of IL-1B. Combined, these results suggest that club cell CREB directly interacts with FOXA2 (Figure 7D), where it has potential modify the transcriptional network responsible for conversion of club cells to goblet cells.

## Discussion

In the current study, we hypothesized that loss of club cell Creb1 would mitigate the pro-mucin effects of IL-1B in mice. Through assaying the entire lung, we found that IL-1B increased mRNA abundance for secreted (*Muc5ac, Muc5b*) and tethered (*Muc1, Muc4*). While loss of club cell Creb1 did not influence these effects of IL-1B, we did observe that loss of club cell Creb1 modified the mRNA abundance of key molecules important for regulated mucin secretion, including *Rab3D, M3R*, and *P2ry2*. All three molecules are known targets of Creb1 (Rouillard et al., 2016).

These observations prompted us to measure goblet cell density under basal and post methacholine stimulated conditions. We found that IL-1B treatment increased detection of goblet cells with neutral mucins in wild type mice post methacholine stimulation, but not in mice with conditional loss of club cell Creb1. Since loss of club cell Creb1 increased *Rab3D* expression, it is possible that mucin secretion was enhanced, perhaps through Rab3D-dependent mechanisms. Prior studies partly support this idea since mice overexpressing Rab3D show enhanced regulated secretion from the pancreatic acinar cells (Ohnishi et al., 1997).

We did not see a major effect of IL-1B or loss of club cell Creb1 on the density of goblet cells expressing acidic mucins under basal or post methacholine stimulation. The apparent selectivity for neutral mucins based upon our histological staining was surprising. We are unsure of the reasons for this, but one interpretation is that IL-1B treated airways in wild type mice have higher ratios of acidic mucins relative to neutral mucins compared to wild type mice treated with vehicle under methacholine stimulated conditions. Indeed, purified mucin secretions from CF airways contain a higher proportion of acidic mucins compared to healthy controls (Xia et al., 2005). Thus, our data appear to be consistent with this finding. Moreover, our data suggest that club cell Creb1 may be an important regulatory molecule that shifts secretion mechanisms.

Repeated administration of IL-1B profoundly modified pulmonary mechanics in response to the secretagogue and smooth muscle contractile agent methacholine, with conditional loss of club cell Creb1 suppressing these effects in mice. Though we hypothesized that increased mucus would produce a parallel increase in airway resistance, which we observed, we were surprised to find that other airway mechanic properties were also impacted. Yet other studies have shown that transient expression of IL-1B induces airway injury and profibrotic responses (Kolb et al., 2001). Lung injury and pulmonary fibrosis increase airway elastance (Manali et al., 2011) and in our study, both airway elastance and tissue elastance were increased in wild type mice treated with IL-1B. Conditional loss of club cell Creb1 suppressed these effects, suggesting that loss of club cell Creb1 may also have implications for airway injury and repair (Park et al., 2021).

We found that IL-1B increased the mRNA expression of *Il1r1* in the lungs of wild type mice, but not in mice with loss of club cell Creb1. *Il1r1* is a target of the steroid receptor coactivator 3 (SRC-3) and activation of SRC requires CREB binding protein (Tanaka et al., 2018). Our results therefore suggest that CREB might play an upstream role in the regulation of the Il1r1. Il1r1 is also a marker of neutrophilic inflammation (Evans et al., 2018), and interestingly, we observed a reduction in the percent of neutrophils in the BAL of mice with conditional loss of club cell Creb1. Whether this is a cause-and-effect relationship, or a simple correlation, is unknown and merits further investigation. Though IL-1B increased *Il1r1* mRNA in the total lung of wild type mice, we did not see a corresponding increase in the percentage of granulocytes in the BAL. Our studies did not delineate which cells contributed to the increased *Il1r1* mRNA. Therefore, it is difficult to predict whether increased *Il1r1* mRNA in the total lung reflects an increase in the cells required for neutrophil recruitment.

We implemented a discovery-driven approach to assess global impacts on inflammation through use of inflammatory-directed PCR arrays in mice. We identified select genes that were differentially expressed as consequence of treatment and/or genotype. One gene of interest, *Ly96*, was decreased in lungs of wild type mice treated with IL-1B but increased in mice treated with IL-1B that had conditional loss of club cell Creb1. Previous work has shown that overexpression of specific isoforms of this gene are protective against airway inflammation (Tumurkhuu et al., 2015). Therefore, it is possible that increased Ly96 in mice with conditional loss of club cell Creb1 has beneficial anti-inflammatory effects.

Finally, we adopted a reductionist approach and studied a human cell line consisting of airway club cells to determine potential mechanisms responsible for the protective effects of loss of club cell Creb1 observed *in vivo*. We corroborated our *in vivo* findings and found that treatment with IL-1B increased expression of CREB in club cells *in vitro*. We also established that the human “club cell-like” line expressed the receptor for IL-1B, and that treatment with IL-1B increased cAMP concentrations in these cells. This finding was consistent with previous data demonstrating a similar increase in cAMP concentrations in response to IL-1B in human myometrial cells (Oger et al., 2002) and work showing IL-1B increases cAMP in human airway epithelia (Gray et al., 2004; Clayton et al., 2005). Therefore, we directly established a link among IL-1B, cAMP, and CREB in human club cells.

Our *in vitro* studies also revealed an important role for club cell CREB in regulating FOXA2 and SPDEF, two critical transcription factors that govern goblet cell expansion. Though prior work suggested that CREB regulates *FOXA2* (Zhang et al., 2005; Baroukh et al., 2007; Everett et al., 2013), such a relationship had not been described for airway cells. Our studies demonstrate that CREB regulates FOXA2 through direct interactions in airway cells. Moreover, our studies corroborated prior work demonstrating a reciprocal relationship between SPDEF and FOXA2 expression (Chen et al., 2009; Chen et al., 2010; Yu et al., 2010; Choi et al., 2020). Our results do not support a direct regulation of SPDEF by CREB, but rather that SPDEF is regulated through CREB-FOXA2 interactions, or other upstream factor(s) activated by CREB. This study opens the door for additional mechanistic studies to define these relationships.

One limitation of our *in vitro* study is that it is unknown whether NCI-H322 cells undergo differentiation to goblet cells *in vitro,* even after stimulation with IL-1B. Though IL-1B–mediated upregulation of MUC5AC in primary differentiated human bronchial epithelial cells occurs at the dose of IL-1B we used in this study (i.e., 10 ng/mL) (Chen et al., 2014), it is possible this dose was not strong enough to upregulate mucins in the NCI-H322 cells. Alternatively, it is possible that NCI-H322 cells require longer exposure to IL-1B or additional factors for conversion to goblet cells *in vitro*.

In summary, our study identified club cell Creb1 as an important modulator of IL-1B-mediated pro-mucin and pro-inflammatory effects. We further determined that human CREB1 regulates genes important for goblet cell expansion through direct, and likely indirect, interactions. It is possible that targeting club cell CREB1 may have therapeutic implications for CF and other airway diseases.

## Data Availability

The raw data supporting the conclusion of this article will be made available by the authors, without undue reservation.
