# Supplementary material for: Club cell CREB regulates the goblet cell transcriptional network and pro-mucin effects of IL-1B
Source: Front Physiol. 2023 Dec 20;14:1323865. doi: 10.3389/fphys.2023.1323865 (PMC10761479; doi:10.3389/fphys.2023.1323865)
Supplement: Supplementary file 1 [file Table4.pdf]

Supplemental Table S4. Statistical data for genes that were differentially expressed in inflammatory-directed lung arrays according to FDR (shown in Figure 5D).

Gene symbol	Comparison that was statistically significant	Fold-change	P Value	FDR
Csf2	Creb1$^{fl/fl}$Scgb1a1^{wt} + VEH vs Creb1$^{fl/fl}$Scgb1a1^{wt} IL-1B	0.499	0.0036	0.0076
	Creb1$^{fl/fl}$Scgb1a1^{cre} + VEH vs Creb1$^{fl/fl}$Scgb1a1^{cre} + IL-1B	0.354	0.0038	0.0499
Il10	Creb1$^{fl/fl}$Scgb1a1^{cre} + VEH vs Creb1$^{fl/fl}$Scgb1a1^{cre} + IL-1B	0.536	0.0018	0.0022
Ly96	Creb1$^{fl/fl}$Scgb1a1^{wt} + VEH vs Creb1$^{fl/fl}$Scgb1a1^{wt} IL-1B	0.391	0.0376	0.0395
	Creb1$^{fl/fl}$Scgb1a1^{cre} + VEH vs Creb1$^{fl/fl}$Scgb1a1^{cre} + IL-1B	-0.593	0.0016	0.0034
	Creb1$^{fl/fl}$Scgb1a1^{wt} IL-1B vs Creb1$^{fl/fl}$Scgb1a1^{cre} + IL-1B	-0.790	<0.0001	0.0001
Mpo	Creb1$^{fl/fl}$Scgb1a1^{wt} + VEH vs Creb1$^{fl/fl}$Scgb1a1^{wt} IL-1B	0.451	0.0085	0.0179
Stat4	Creb1$^{fl/fl}$Scgb1a1^{wt} + VEH vs Creb1$^{fl/fl}$Scgb1a1^{cre} + VEH	0.485	0.0047	0.0245
Tlr8	Creb1$^{fl/fl}$Scgb1a1^{cre} + VEH vs Creb1$^{fl/fl}$Scgb1a1^{cre} + IL-1B	-0.595	0.0005	0.0017
