# Supplementary material for: Club cell CREB regulates the goblet cell transcriptional network and pro-mucin effects of IL-1B
Source: Front Physiol. 2023 Dec 20;14:1323865. doi: 10.3389/fphys.2023.1323865 (PMC10761479; doi:10.3389/fphys.2023.1323865)
Supplement: Supplementary file 2 [file Table1.pdf]

Supplemental Table S1. Primer pairs used for real-time and *end-point PCR analyses.

Gene Symbol	Gene	GenBank accession	Sequence of Forward (5'-3') and Reverse (3'-5') primers	Amplicon (bp)
Il1r1	interleukin 1 receptor, type I	NM_008362.2	5' GGAGAAGATACCATCGAGGTTAC<br>3' CTTCCTGGATGAGAGCATTGT	148
Muc5b	mucin 5, subtype B, tracheobronchial	NM_028801.2	5' ACATCCTGACCAAGAAATGTGC<br>3' GACAAGGGCATCTGCGTAAAG	190
Muc5ac	mucin 5, subtypes A and C, tracheobronchial/gastric	NM_010844.3	5' GTGGTGGAAACTGACATTGG<br>3' CATCAAAGTTCCCACACAGG	115
**Creb1*	cAMP responsive element binding protein 1	NM_133828.2	5' GAGAGAATAAAACTCCAGCGAGA<br>3' GTAGGAAGGCCTCCTTGAAAG	436
Cre	Cre recombinase	-	5' TGCCTGCATTACCGGTCG<br>3'GCATAACCAGTGAAACAGCATTGCTG	321
Rab3D	member RAS oncogene family (Rab3d)	NM_031874.5	5' CGAGATCCACGTGTCGGAAG<br>3' CACTAGCGGATGCCATCTCA	312
P2y2	Purinergic receptor P2Y, G-protein coupled receptor 2	NM_008773.4	5'AGCCCATTACGTGACTGTCCCGAG<br>3' CCGGAGGACTCCGAGATCA	128
Muc1	mucin 1, transmembrane	NM_013605.2	5'AGCATCAAGTTCAGGTCAGGC<br>3' GACTTCACGTCAGAGGCACTAA	85
Muc4	mucin 4	NM_080457.5	*5'CCTTCACTGATAACCGCTGCTT*<br>3' *GCGGAGGCATTTTCATCCT*	115
M3r	cholinergic receptor, muscarinic 3	NM_033269.4	5'CACGAGCGAACCTGAGGAC<br>3'ATGCCATTGCTGGTCATATCTGG	162
Actb	actin, beta	NM_007393.5	5' CTGTGGCATCCATGAAACTACA<br>3' GTAATCTCCTTCTGCATCCTGTCA	141
RPL13A	ribosomal protein L13a	NM_012423.4	5' GGCCCCTACCACTTCCG<br>3' ACTGCCTGGTACTTCCA	251

SPDEF	SAM pointed domain containing ETS transcription factor	NM_012391.3	5’ CCAGTGGCCAACCTGAGTG<br>3’ TGGCGGCTGTGTCTGTTAG	185
BDNF	brain derived neurotrophic factor	NM_170735.6	5’ CTCGTGACAGCATGAGCAGAG<br>3’ GACATGCAGTGTTTCCCCCAA	157
CREB1	cAMP responsive element binding protein 1	NM_004379.5	5’ CAGTATATTGCCATTACCCAGGG<br>3’ CAGTGGTCTGTGCATACTGTAG	145
FOXA2	forkhead box A2	NM_021784.5	5’ ACTATGCAGAGCCCGAGG<br>3’ CACGTACGACGACATGTTCA	155
ILIR1	interleukin 1 receptor, type I	NM_000877.4	5’ AGCAAGACACCTGTATCTACAGAA<br>3’ TGCATTATAACATAAGTTAGGCTCATTCTC	188
*GAPDH**	glyceraldehyde-3-phosphate dehydrogenase	2597	5’ AGGTGAAGGTCGGAGTCAA<br>3’ CCCATACGACTGCAAAGACC	117
*FOXA2**	forkhead box A2	3170	5’ GGTGTGAGCTGATTATTCAAATGG<br>3’ GACAAGTGCCGCAGTGA	107
*SPDEF**	SAM pointed domain containing ETS transcription factor	25803	5’ CCGCAACCAGTCTCAGG<br>3’ CGCACCACCAGGATAAAGA	111

* Primers used for endpoint PCR after ChIP assays.
