# Supplementary material for: Club cell CREB regulates the goblet cell transcriptional network and pro-mucin effects of IL-1B
Source: Front Physiol. 2023 Dec 20;14:1323865. doi: 10.3389/fphys.2023.1323865 (PMC10761479; doi:10.3389/fphys.2023.1323865)
Supplement: Supplementary file 3 [file Table2.pdf]

Supplemental Table S2. Descriptive statistics for inflammatory-directed lung array data in Creb1$^{fl/fl}$Scgb1a1^{cre} or Creb1$^{fl/fl}$Scgb1a1^{wt} mice that received vehicle (VEH) or interleukin 1β (IL-1B).

	Creb1$^{fl/fl}$Scgb1a1^{wt} + VEH			Creb1$^{fl/fl}$Scgb1a1^{wt} + IL-1B			Creb1$^{fl/fl}$Scgb1a1^{cre} + VEH			Creb1$^{fl/fl}$Scgb1a1^{cre} + IL-1B		
**Gene**	Mean	SEM	N	Mean	SEM	N	Mean	SEM	N	Mean	SEM	N
Apcs	1	0.22	6	0.85	0.13	6	0.81	0.23	6	1.05	0.22	6
C3	1	0.04	6	0.82	0.03	6	0.81	0.08	6	0.98	0.05	6
C5ar1	1	0.12	6	0.92	0.12	6	0.73	0.05	6	1.01	0.11	6
Casp1	1	0.07	6	0.93	0.01	6	0.79	0.07	6	1.02	0.1	6
Ccl12	1	0.11	6	0.85	0.05	6	1.11	0.22	6	0.99	0.16	6
Ccl5	1	0.13	6	1.02	0.09	6	0.94	0.06	6	0.95	0.07	6
Ccr4	1	0.09	6	1.07	0.13	6	0.95	0.13	6	0.99	0.12	6
Ccr5	1	0.05	6	0.97	0.06	6	0.91	0.14	6	0.98	0.06	6
Ccr6	1	0.14	6	1.01	0.12	6	0.66	0.1	6	0.91	0.08	6
Ccr8	1	0.09	6	1.21	0.21	6	0.96	0.12	6	1.1	0.13	6
Cd14	1	0.09	6	0.93	0.1	6	1.05	0.05	6	1	0.05	6
Cd4	1	0.07	6	0.99	0.04	6	0.84	0.11	6	0.97	0.08	6
Cd40	1	0.05	6	0.99	0.07	6	0.97	0.03	6	0.99	0.04	6
Cd40lg	1	0.14	6	0.9	0.07	6	0.87	0.13	6	0.82	0.11	6
Cd80	1	0.08	6	1.09	0.09	6	0.82	0.11	6	1.07	0.14	6
Cd86	1	0.05	6	1.06	0.05	6	0.92	0.06	6	0.97	0.07	6
Cd8a	1	0.06	6	0.95	0.07	6	0.78	0.1	6	0.91	0.09	6
Crp	1	0.17	6	0.77	0.09	6	0.85	0.15	6	0.98	0.17	6
Csf2	1	0.28	6	0.5	0.06	6	0.84	0.26	6	0.49	0.08	6
Cxcl10	1	0.14	6	0.78	0.1	6	0.77	0.08	6	0.86	0.11	6
Cxcr3	1	0.07	6	0.97	0.06	6	1.01	0.08	6	0.95	0.04	6
Ddx58	1	0.06	6	0.93	0.03	6	0.83	0.12	6	1.01	0.06	6
Fasl	1	0.12	6	0.98	0.11	6	0.92	0.16	6	1.01	0.14	6

Foxp3	1	0.06	6	1.12	0.11	6	0.93	0.19	6	1.1	0.11	6
Gata3	1	0.23	6	0.71	0.08	6	0.76	0.16	6	0.91	0.18	6
H2-Q10	1	0.13	6	0.9	0.13	6	0.58	0.05	6	0.86	0.07	6
H2-T23	1	0.09	6	0.97	0.05	6	0.95	0.07	6	0.97	0.07	6
Icam1	1	0.07	6	0.97	0.04	6	1.04	0.04	6	1.11	0.04	6
Ifna2	1	0.18	6	0.69	0.07	6	0.75	0.12	6	0.86	0.15	6
Ifnar1	1	0.1	6	0.99	0.08	6	0.95	0.06	6	1.06	0.1	6
Ifnb1	1	0.21	6	0.75	0.1	6	0.69	0.1	6	0.97	0.18	6
Ifng	1	0.13	6	1.39	0.18	6	1.15	0.11	6	1.06	0.12	6
Ifngr1	1	0.04	6	1.03	0.06	6	1.1	0.04	6	1.1	0.05	6
Il10	1	0.16	6	0.97	0.28	6	1.53	0.16	6	0.99	0.17	6
Il17a	1	0.16	6	0.62	0.08	6	0.78	0.15	6	0.8	0.16	6
Il18	1	0.06	6	1.05	0.09	6	0.97	0.04	6	1.05	0.04	6
Il1a	1	0.11	6	0.91	0.06	6	0.85	0.1	6	0.93	0.12	6
Il1b	1	0.1	6	0.99	0.14	6	0.61	0.09	6	0.84	0.08	6
Il1r1	1	0.09	6	0.96	0.05	6	0.95	0.1	6	1.04	0.06	6
Il2	1	0.15	6	0.8	0.07	6	0.86	0.14	6	0.82	0.14	6
Il23a	1	0.28	6	0.95	0.21	6	0.94	0.17	6	0.97	0.21	6
Il4	1	0.13	6	0.81	0.08	6	0.82	0.09	6	0.85	0.09	6
Il5	1	0.15	6	1	0.07	6	0.98	0.16	6	0.99	0.09	6
Il6	1	0.11	6	0.88	0.11	6	0.97	0.2	6	0.82	0.17	6
Irak1	1	0.06	6	0.9	0.07	6	1.01	0.05	6	1.02	0.1	6
Irf3	1	0.06	6	1.13	0.06	6	1.12	0.05	6	1.03	0.06	6
Irf7	1	0.09	6	0.9	0.07	6	0.81	0.04	6	0.99	0.17	6
Itgam	1	0.14	6	0.86	0.13	6	0.59	0.08	6	0.95	0.09	6
Jak2	1	0.04	6	1.01	0.05	6	0.96	0.07	6	1.05	0.04	6
Ly96	1	0.28	5*	0.61	0.1	5*	0.81	0.24	5*	1.4	0.56	5*
Lyz2	1	0.08	6	1.02	0.07	6	1.13	0.16	6	1.01	0.06	6
Mapk1	1	0.05	6	1.11	0.06	6	1.02	0.06	6	1.07	0.09	6

Mapk8	1	0.05	6	1.2	0.08	6	0.9	0.08	6	1.17	0.08	6
Mbl2	1	0.17	6	0.71	0.07	6	0.88	0.11	6	0.91	0.16	6
Mpo	1	0.23	6	0.55	0.18	6	0.38	0.1	6	0.67	0.14	6
Mx1	1	0.1	6	0.82	0.05	6	0.73	0.12	6	0.9	0.14	6
Myd88	1	0.05	6	0.95	0.03	6	0.92	0.03	6	0.98	0.06	6
Nfkb1	1	0.08	6	0.89	0.08	6	0.94	0.11	6	1.06	0.08	6
Nfkbia	1	0.02	6	1.06	0.07	6	1.21	0.1	6	1.18	0.13	6
Nlrp3	1	0.09	6	0.88	0.05	6	0.66	0.1	6	0.83	0.03	6
Nod1	1	0.05	6	0.97	0.03	6	0.95	0.1	6	1	0.04	6
Nod2	1	0.12	6	0.9	0.05	6	0.85	0.08	6	0.96	0.09	6
Rag1	1	0.37	6	0.58	0.15	6	0.84	0.39	6	0.9	0.24	6
Rorc	1	0.04	6	1.05	0.08	6	1.17	0.09	6	1.08	0.05	6
Slc11a1	1	0.06	6	0.94	0.06	6	0.84	0.05	6	1.03	0.03	6
Stat1	1	0.35	6	0.64	0.1	6	0.57	0.06	6	0.59	0.06	6
Stat3	1	0.08	6	0.88	0.06	6	0.82	0.06	6	1.05	0.1	6
Stat4	1	0.14	6	0.61	0.09	6	0.51	0.09	6	0.72	0.16	6
Stat6	1	0.04	6	0.94	0.04	6	0.92	0.06	6	0.98	0.06	6
Tbx21	1	0.11	6	0.96	0.06	6	0.79	0.13	6	1.07	0.06	6
Ticam1	1	0.11	6	0.92	0.07	6	0.85	0.1	6	1.08	0.12	6
Tlr1	1	0.16	6	1.1	0.11	6	0.86	0.11	6	1.03	0.12	6
Tlr2	1	0.07	6	1.1	0.05	6	0.87	0.05	6	1	0.03	6
Tlr3	1	0.21	6	0.92	0.14	6	0.81	0.1	6	0.97	0.21	6
Tlr4	1	0.08	6	0.81	0.08	6	0.79	0.08	6	1.21	0.3	6
Tlr5	1	0.13	6	1.03	0.15	6	0.93	0.13	6	1.29	0.23	6
Tlr6	1	0.08	6	0.96	0.08	6	0.77	0.06	6	0.96	0.06	6
Tlr7	1	0.05	6	1.01	0.05	6	0.69	0.08	6	0.95	0.06	6
Tlr8	1	0.12	6	0.93	0.23	6	0.83	0.18	6	1.42	0.29	6
Tlr9	1	0.07	6	0.95	0.11	6	0.82	0.12	6	0.92	0.08	6
Tnf	1	0.07	6	0.94	0.13	6	0.73	0.1	6	0.82	0.06	6

Traf6	1	0.03	6	1.05	0.03	6	1.03	0.08	6	1.08	0.04	6
Tyk2	1	0.04	6	0.98	0.02	6	0.93	0.1	6	1.01	0.06	6

**n = 5 for Ly96 because each group had a single mouse that was below detection*
