# Supplementary material for: Club cell CREB regulates the goblet cell transcriptional network and pro-mucin effects of IL-1B
Source: Front Physiol. 2023 Dec 20;14:1323865. doi: 10.3389/fphys.2023.1323865 (PMC10761479; doi:10.3389/fphys.2023.1323865)
Supplement: Supplementary file 4 [file Image4.pdf]

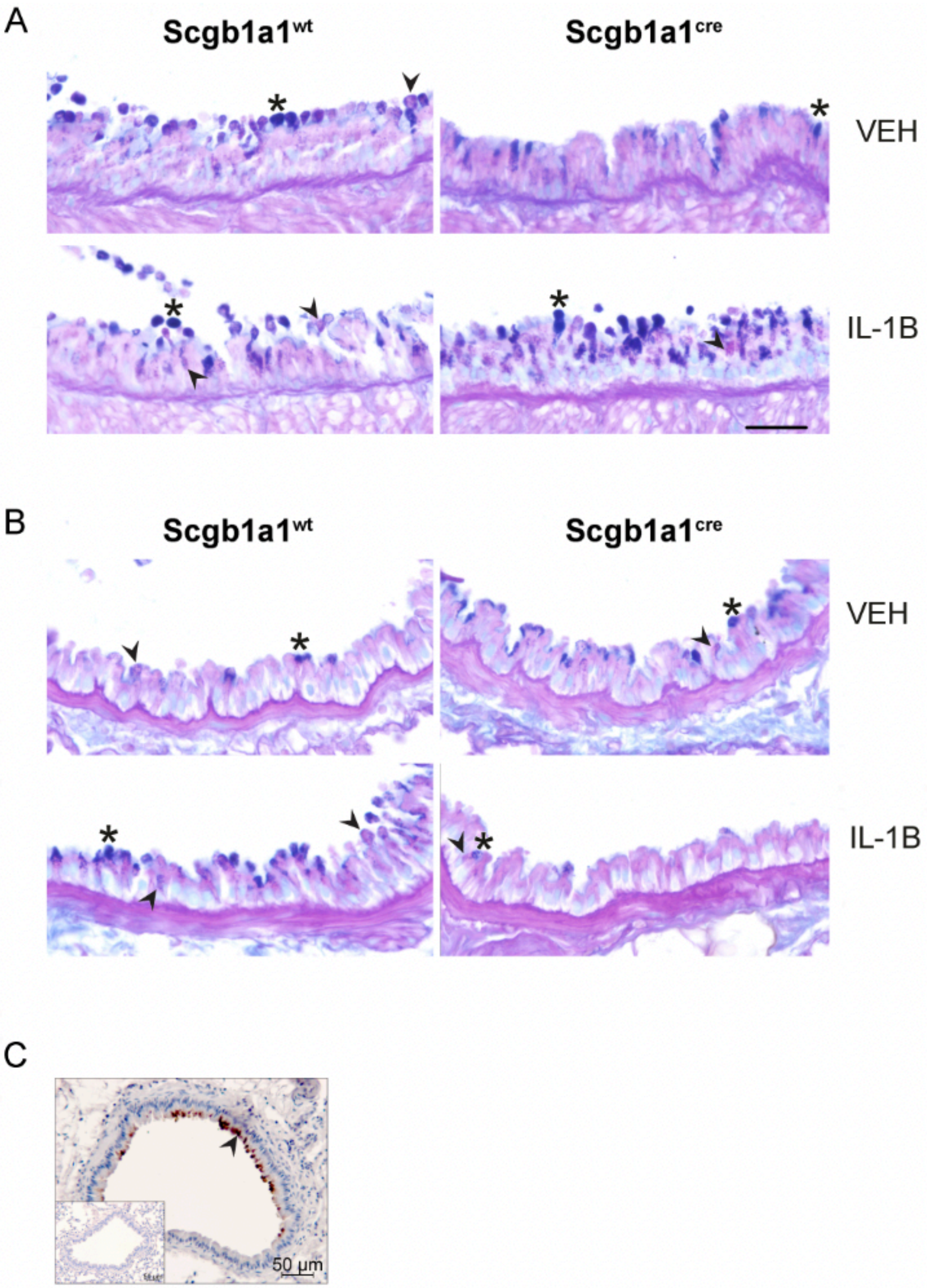
A
Scgb1a1wt
Scgb1a1cre
VEH
IL-1B
B
Scgb1a1wt
Scgb1a1cre
VEH
IL-1B
C
50 µm

Supplemental Figure S4. Conditional loss of club cell Creb1 and mucin secretion properties. Representative images of Alcian Blue/Periodic acid–Schiff (PAS) staining in central airways from Creb1$^{fl/fl}$Scgb1a1^{cre} or Creb1$^{fl/fl}$Scgb1a1^{wt} mice under basal **(A)** and post methacholine stimulated **(B)** conditions. Arrow indicates an example of PAS-positive cells stained purple (neutral mucins). Asterisks represent examples of Alcian Blue- positive cells (acidic mucins). Scale bar in panel A is 50 μm and applies to panel B. (**C**) Representative image of Muc5b immunohistochemistry in central airway post methacholine stimulation. Arrow indicates an example of Muc5b-positive cell. Inset shows no primary control. Scale bar is 50 μm.
