# Supplementary material for: Club cell CREB regulates the goblet cell transcriptional network and pro-mucin effects of IL-1B
Source: Front Physiol. 2023 Dec 20;14:1323865. doi: 10.3389/fphys.2023.1323865 (PMC10761479; doi:10.3389/fphys.2023.1323865)
Supplement: Supplementary file 5 [file Image2.pdf]

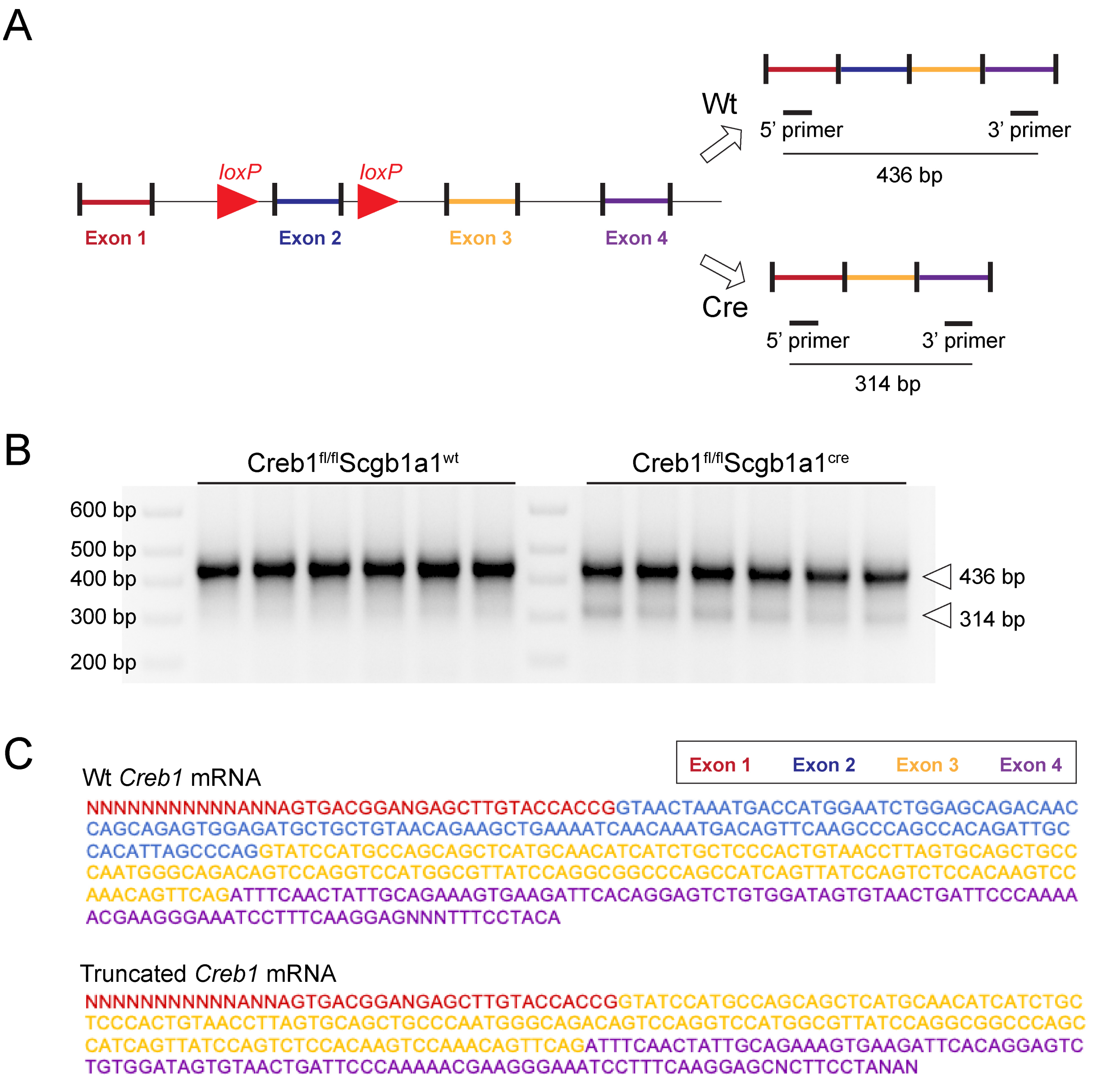


**Supplemental Figure S2. Cre–mediated recombination in mouse lung.** (**A**) *Creb1* gene structure and recombination strategy. (**B**) cDNA from lung RNA samples was used as template for PCR reactions to confirm the presence of truncated mRNA transcribed by Cre/lox-specific excision of *Creb1* exon 2 in Creb1$^{fl/fl}$Scgb1a1$^{cre}$ mice in total lung homogenates. The 436 bp product represents the mRNA transcribed from the non-recombined *Creb1*. A 314 bp fragment, which was detected in Creb1$^{fl/fl}$Scgb1a1$^{cre}$ samples, represents the truncated *Creb1* mRNA when

excision of exon 2 occurred. (**C**) Sequencing of the 314 bp fragment confirmed lox-specific recombination.
