# Supplementary material for: Club cell CREB regulates the goblet cell transcriptional network and pro-mucin effects of IL-1B
Source: Front Physiol. 2023 Dec 20;14:1323865. doi: 10.3389/fphys.2023.1323865 (PMC10761479; doi:10.3389/fphys.2023.1323865)
Supplement: Supplementary file 6 [file Image3.pdf]

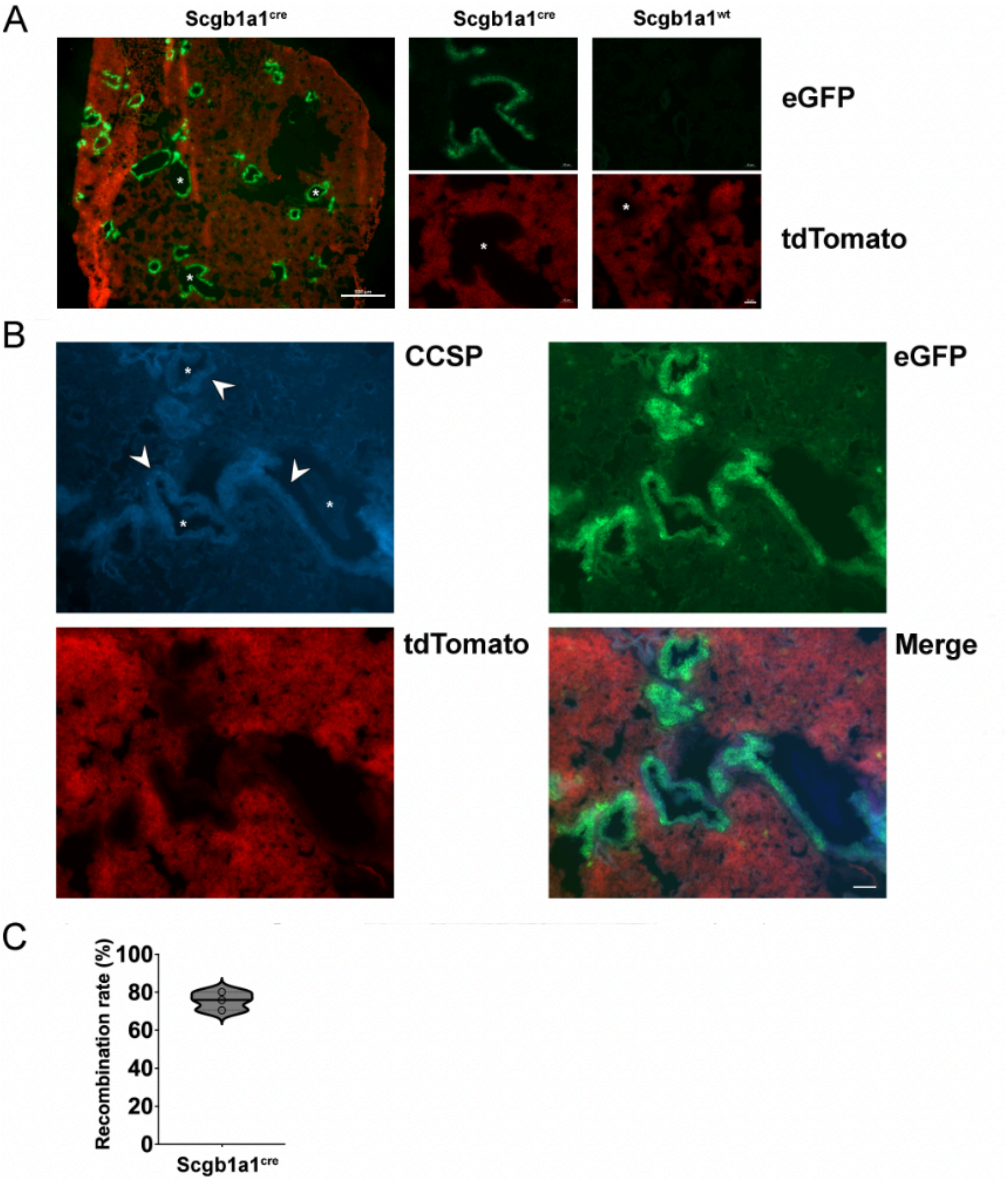

Supplemental Figures S3. **Additional assessment of Cre–mediated recombination in mouse lung.** (**A**) Low power magnification image of lung section demonstrating Cre activity in ROSA$^{mT/mG}$ Creb1$^{fl/fl}$Scgb1a1^{cre} mice provided two injections of tamoxifen as detailed in methods. Widespread expression of membrane-located tdTomato is replaced with membrane-

located eGFP due to Scgb1a-driven cre expression and recombination. Asterisks represent examples of airway lumens. Scale bar is 500 µm. $ROSA^{mT/mG}$ $Creb1^{fl/fl}Scgb1a1^{wt}$ are shown as a control. Scale bar is 50 µm. (**B**) Image of mouse lung from $ROSA^{mT/mG}$ $Creb1^{fl/fl}Scgb1a1^{cre}$ immuno-labeled with club cell secretory protein (CCSP) in blue. Arrows represent examples of airway epithelia and asterisks represent examples of airway lumens. Subsequent panels show separate channels for eGFP, tdTomato, and the merged image. Scale bar is 50 µm. (**C**) Violin plot showing quantification of the percent of CCSP-labeled cells (blue) that were also expressing eGFP in the $ROSA^{mT/mG}$ $Creb1^{fl/fl}Scgb1a1^{cre}$ mice. This percent is considered the recombination rate. n = 3 mice. The median is thick center line; the quartiles are shown as thinner dashed lines. Individual points represent the data from a single mouse. Abbreviations: WT, wild type; $Scgb1a1^{cre}$, club cell promoter driving CRE recombinase; CCSP, club cell secretory protein.
