# Supplementary material for: Club cell CREB regulates the goblet cell transcriptional network and pro-mucin effects of IL-1B
Source: Front Physiol. 2023 Dec 20;14:1323865. doi: 10.3389/fphys.2023.1323865 (PMC10761479; doi:10.3389/fphys.2023.1323865)
Supplement: Supplementary file 7 [file Table3.pdf]

Supplemental Table S3. Descriptive statistics for cell cycle-directed array data in H322 cells that received vehicle (VEH) or interleukin 1β (IL-1B) for four days.

	VEH			IL-1B		
**Gene**	Mean	SEM	N	Mean	SEM	N
ABL1	1	0.06	3	0.87	0.02	3
ANAPC2	1	0.04	3	0.90	0.05	3
ATM	1	0.15	3	1.04	0.06	3
ATR	1	0.75	3	0.86	0.52	3
AURKA	1	0.11	3	0.82	0.04	3
AURKB	1	0.06	3	1.02	0.05	3
BCCIP	1	0.10	3	0.84	0.01	3
BCL2	1	0.05	3	0.85	0.02	3
BRCA1	1	0.11	3	2.48	1.23	3
CASP3	1	0.02	3	0.82	0.08	3
CCNA2	1	0.13	3	0.90	0.09	3
CCNB1	1	0.48	3	0.60	0.04	3
CCND1	1	0.11	3	0.83	0.03	3
CCND2	1	0.15	3	0.56	0.07	3
CCND3	1	0.07	3	0.83	0.04	3
CCNE1	1	0.04	3	0.89	0.05	3
CCNF	1	0.14	3	1.14	0.14	3
CCNG1	1	0.20	3	1.29	0.28	3
CCNG2	1	0.18	3	0.74	0.12	3
CCNH	1	0.04	3	0.95	0.05	3
CCNT1	1	0.02	3	0.88	0.04	3
CDC16	1	0.33	3	0.71	0.09	3

CDC20	1	0.06	3	0.90	0.04	3
CDC25A	1	0.03	3	0.93	0.02	3
CDC25C	1	0.10	3	0.83	0.03	3
CDC34	1	0.07	3	0.84	0.03	3
CDC6	1	0.08	3	0.75	0.03	3
CDK1	1	0.19	3	1.00	0.00	3
CDK2	1	0.08	3	0.83	0.01	3
CDK4	1	0.07	3	0.90	0.01	3
CDK5R1	1	0.09	3	0.78	0.01	3
CDK5RAP1	1	0.10	3	0.94	0.05	3
CDK6	1	0.01	3	0.86	0.02	3
CDK7	1	0.01	3	1.29	0.10	3
CDK8	1	0.07	3	0.86	0.03	3
CDKN1A	1	0.17	3	0.95	0.16	3
CDKN1B	1	0.09	3	0.80	0.06	3
CDKN2A	1	0.35	3	0.92	0.04	3
CDKN2B	1	0.60	3	1.51	0.67	3
CDNK3	1	0.15	3	0.91	0.05	3
CHEK2	1	0.16	3	0.83	0.11	3
CKS1B	1	0.05	3	0.89	0.02	3
CKS2	1	0.08	3	0.96	0.10	3
CUL2	1	0.05	3	0.88	0.02	3
CUL3	1	0.06	3	0.92	0.04	3
E2F1	1	0.09	3	0.81	0.05	3
E2F4	1	0.02	3	0.87	0.04	3
GADD45A	1	0.07	3	0.93	0.12	3
GTSE1	1	0.06	3	0.84	0.12	3
HUS1	1	0.07	3	0.80	0.05	3
KNTC1	1	0.09	3	0.84	0.04	3

KPNA2	1	0.12	3	0.97	0.10	3
MAD2L1	1	0.09	3	0.87	0.04	3
MAD2L2	1	0.05	3	0.89	0.03	3
MCM2	1	0.06	3	0.89	0.04	3
MCM3	1	0.08	3	0.89	0.00	3
MCM4	1	0.11	3	0.87	0.07	3
MCM5	1	0.07	3	0.91	0.06	3
MDM2	1	0.12	3	0.75	0.09	3
MKI67	1	0.15	3	1.15	0.13	3
MNAT1	1	0.08	3	1.06	0.06	3
MRE11	1	0.08	3	0.88	0.28	3
NBN	1	0.07	3	0.98	0.11	3
RAD17	1	0.06	3	0.83	0.11	3
RAD9A	1	0.09	3	0.86	0.04	3
RB1	1	0.05	3	0.83	0.02	3
RBBP8	1	0.04	3	0.69	0.05	3
RBL1	1	0.04	3	0.82	0.01	3
RBL2	1	0.11	3	0.74	0.02	3
SERTAD1	1	0.05	3	1.00	0.13	3
SKP2	1	0.06	3	0.79	0.06	3
STMN1	1	0.08	3	0.95	0.01	3
TFDP1	1	0.12	3	1.25	0.30	3
TFDP2	1	0.11	3	0.92	0.10	3
TP53	1	0.04	3	0.87	0.02	3
WEE1	1	0.32	3	1.57	0.32	3
