# Supplementary material for: Club cell CREB regulates the goblet cell transcriptional network and pro-mucin effects of IL-1B
Source: Front Physiol. 2023 Dec 20;14:1323865. doi: 10.3389/fphys.2023.1323865 (PMC10761479; doi:10.3389/fphys.2023.1323865)
Supplement: Supplementary file 8 [file Image1.pdf]

LIST OF SUPPLEMENTARY MATERIALS

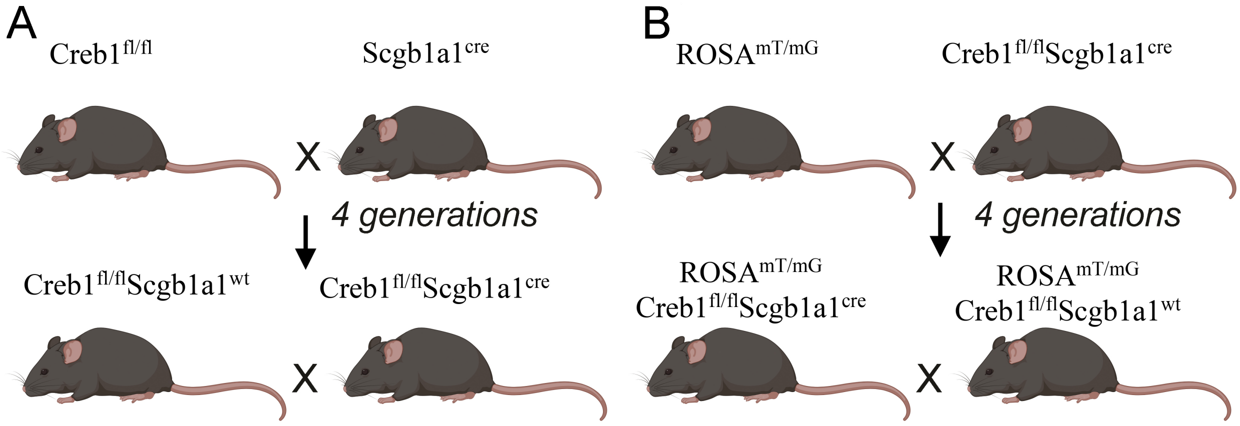

Supplemental Figure S1. Simplified breeding scheme. (**A**) Mice with floxed *Creb1* genes (Creb1$^{fl/fl}$) were bred to mice with an inducible Cre under the *Scgb1a1* promoter. The University of Florida Rodent Models breeding core performed appropriate crossings for four generations to obtain desired genotypes of mice that were Creb1$^{fl/fl}$Scgb1a1^{cre} and Creb1$^{fl/fl}$Scgb1a1^{wt}. These mice were then bred for continued generations and studied. (**B**) Mice with Creb1$^{fl/fl}$Scgb1a1^{cre} were bred to mice with ROSA$^{mT/mG}$ transgenes. The University of Florida Rodent Models breeding core performed appropriate crossings for four generations to obtain desired genotypes of mice that were ROSA$^{mT/mG}$ Creb1$^{fl/fl}$Scgb1a1^{cre} and ROSA$^{mT/mG}$ Creb1$^{fl/fl}$Scgb1a1^{wt}. These mice were then bred for continued generations and studied. Abbreviations: WT, wild type; Scgb1a1^{cre}, club cell promoter driving CRE recombinase.
